# Synthesis of Novel C-2- or C-15-Labeled BODIPY—Estrone Conjugates

**DOI:** 10.3390/molecules23040821

**Published:** 2018-04-03

**Authors:** Ildikó Bacsa, Csilla Konc, Anna Boglárka Orosz, Gábor Kecskeméti, Réka Rigó, Csilla Özvegy-Laczka, Erzsébet Mernyák

**Affiliations:** 1 Department of Organic Chemistry, University of Szeged, Dóm tér 8, H-6720 Szeged, Hungary; bacsa.ildike@gmail.com (I.B.); koncsi11@gmail.com (C.K.); oabogi@gmail.com (A.B.O.); 2 Department of Medicinal Chemistry, University of Szeged, Dóm tér 8, H-6720 Szeged, Hungary; kecskemeti.gabor@med.u-szeged.hu; 3 Membrane protein research group, Institute of Enzymology, Research Centre for Natural Sciences, Hungarian Academy of Sciences, Magyar tudósok körútja 2, H-1117 Budapest, Hungary; rigo.reka@ttk.mta.hu (R.R.); laczka.csilla@ttk.mta.hu (C.Ö.-L.)

**Keywords:** BODIPY, estrone, CuAAC, fluorescent labeling, estrone conjugates

## Abstract

Novel BODIPY–estrone conjugates were synthesized via Cu(I)-catalyzed azide–alkyne cycloaddition (CuAAC). Estrone-alkynes or an estrone-azide as starting compounds were synthesized *via* Michael addition or Sonogashira reaction as key steps. Fluorescent dyes based on BODIPY-core were provided by azide or alkyne functional groups. Fluorescent labeling of estrone was efficiently achieved at the C-2 or C-15 position. The newly-elaborated coupling procedures might have a broad applicability in the synthesis of fluorescent-labeled estrone conjugates suitable for biological assays.

## 1. Introduction

**Figure 1:**
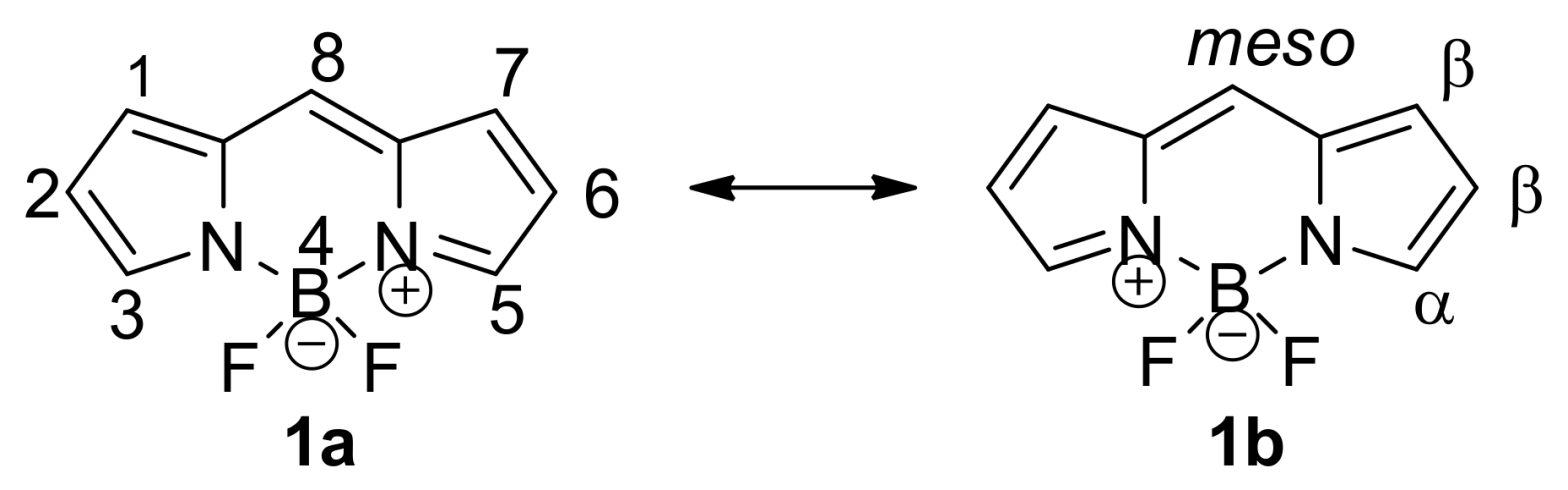
The core structure of 4,4-difluoro-4-bora-3a,4a-diaza-*s*-indacenes (BODIPY) dyes.

4,4-Difluoro-4-bora-3a,4a-diaza-*s*-indacenes (BODIPY, **1**, Figure 1.) are strongly UV-absorbing small dyes with characteristic spectroscopic properties [1,2]. They emit relatively sharp fluorescence peaks with high quantum yields and possess excellent photostability. Additionally, they are stable to physiological conditions owing to their special behavior concerning their insensitivity to pH and the polarity of their environment. Their fluorescence characteristics can be modulated by the directed chemical tuning of the chromophore. An unambiguous trend toward red-shifted absorption and emission maxima with increased substitution at the 1-, 3-, 5- and/or 7-positions is observed. However, alkylation or arylation at the *meso* position has no substantial effect on the absorption and emission wavelengths. These dyes are extensively used in biomolecule labeling owing to their beneficial properties [3,4,5,6,7,8]. Nevertheless, their biological application is limited because of the limited water solubility and emission wavelength (under 600 nm).

The data from the literature reveals several synthetic strategies for the preparation of BODIPY dyes [1,2]. The dipyrrolylmethene core might be constructed from aromatic aldehydes and pyrroles and the subsequent oxidation of the resulting dipyrrolylmethane [7]. After the preparation of the heterocyclic core, complexation with boron leads to the desired BODIPY derivative. In order to obtain the appropriately substituted compound at the *meso* position, a reasonable choice of the aromatic aldehyde is needed. With the aim of avoiding the oxidation step, the dipyrrolylmethene core is usually synthesized via the condensation of acyl chlorides with pyrroles [9,10]. In the latter case, the *meso* substituent is derived from the acyl chloride, thus, the directed selection of the appropriate acyl chloride is crucial. In the case of attaching the BODIPY dye to a biomolecule, the feasibility of the introduction of the desired functional group onto the BODIPY core is of particular interest. The coupling mode of the dye to a biomolecule might be determined with regard to its biological behavior. The development of imaging probes to monitor the mechanism of action of biologically active compounds in living systems is of great interest in medicinal chemistry.

Estrogens belong to a class of natural steroids which possess hormonal activity. However, their chemical modifications may lead to biologically active estrone derivatives lacking hormonal behavior [11,12]. Certain synthetic estrone-derived compounds are described as antitumoral agents, but the mechanism of their action is mostly unknown [11]. In order to investigate and monitor their mechanism, their labeling is essential. Enzymatic or receptorial assays are mainly based on radioisotope labeling. Nowadays, there is a need for the replacement of the harmful radioactive methods for greener and friendlier fluorescent ones. However, to the best of our knowledge, there is no reported general use of BODIPY-labeled estrone derivatives in fluorescent biological assays. BODIPY–estrone conjugates are rarely described in the literature [13]. The compounds known presently are labeled at positions 3-, 7α-, or 17α-, mainly affecting the two oxygen functionalities of the steroid. The coupling procedures involved amide formation, olefin metathesis, the Sonogashira reaction, or the S_N_Ar-type reaction [13]. Certain 7α-conjugates seemed to be able to bind to the estrogen receptor alpha; thus, they might be used in fluorescent receptorial assays. Not only the position of labeling, but the nature of the linking group and the spacing between the two moieties might also have a substantial influence on the biological behavior. There exist BODIPY–estrone conjugates labeled at the 17α-position coupled via Cu(I)-catalyzed azide–alkyne click-reaction (CuAAC) [14]. This is a powerful and convenient route for the conjugation of two molecular entities via metabolically stable triazole linker [15,16]. Click chemistry is emphasized by its high efficiency and tolerance toward several functional groups [15,16].

## 2. Results and Discussion

**Figure 2:**
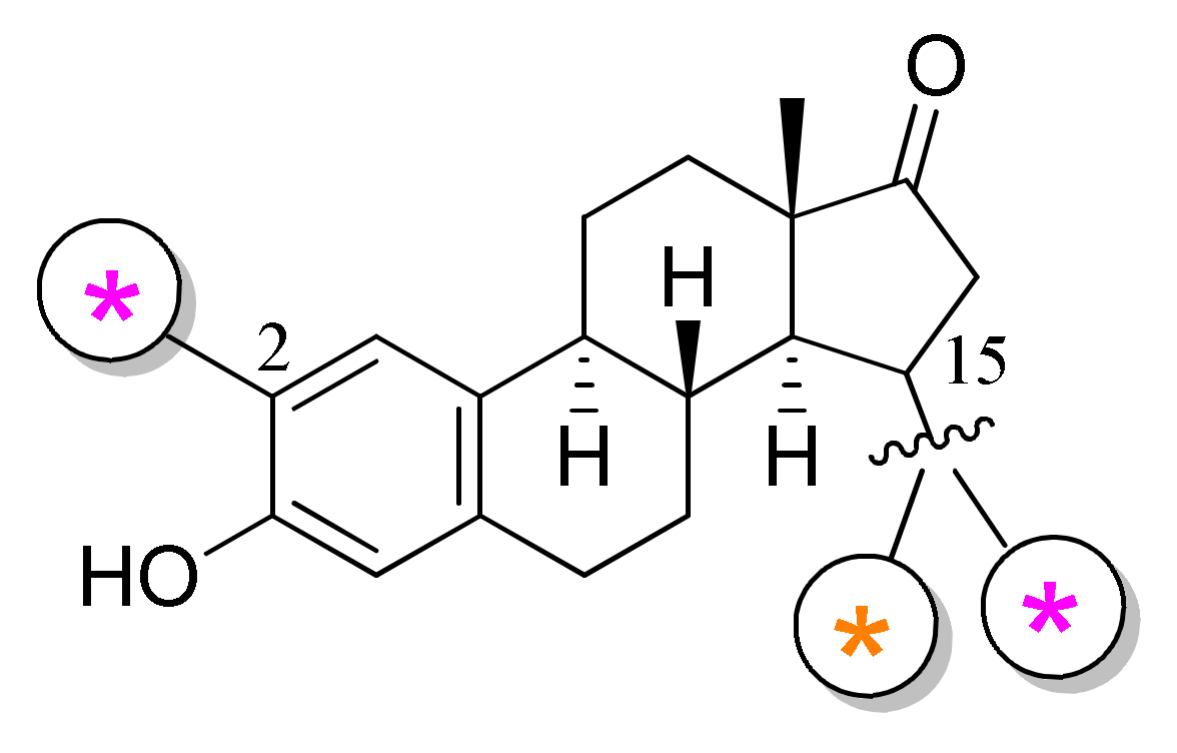
The fluorescent labeling at positions C-2 and C-15 of estrone.

Here we aimed to synthesize BODIPY–estrone conjugates without transforming the two oxygen functionalities of the steroid and by establishing a chemically and metabolically stabile attaching moiety. Therefore, we chose the positions C-2 or C-15 for labeling, thereby avoiding covalent modifications at the C-3 and C-17 positions (Figure 2). Estrone and its 17β-hydroxy counterpart display strong secondary interactions with the enzyme or receptor targets through their keto or hydroxy groups [17,18]. The blocking of these groups may result in biological behavior different than that of estrone or 17β-estradiol. However, all conjugations at sites different than the main functionalities might be of value. According to the literature, there are several 2- or 15-substituted estrone derivatives that are biologically active steroids, displaying important activities such as the antitumoral effect [12,19,20,21,22]. It is known that certain derivatives act through different protein targets, including tubulin or enzymes involved in estrogen biosynthesis [11,12,19,20,21,22]. The fluorescent labeling of compounds potentially interacting with the above-mentioned proteins might have high biological relevance.

Recent advances in chemical synthesis techniques, in particular, in cross coupling and conjugation methods, allow for the accomplishment of reactions which could formerly not be achieved. Concerning the mode of conjugation, clicking via CuAAC reaction is one of the most feasible and effective strategies [15,16,23]. On the basis of our longstanding experience [24,25,26,27,28,29,30,31] in developing steroidal azides and alkynes suitable for the formation of biologically active triazolyl conjugates, here, we chose the CuAAC reaction for attaching the dye to the steroid. An added advantage of the presence of this heterocycle on a steroid core is due to its behavior being similar to that of a peptide bond [32]. Nevertheless, there is a great challenge in the labeling process concerning the establishment of the best reaction sequence after taking into account the reactivity and the sensitivity of the already introduced functional groups.

**Scheme 1:**
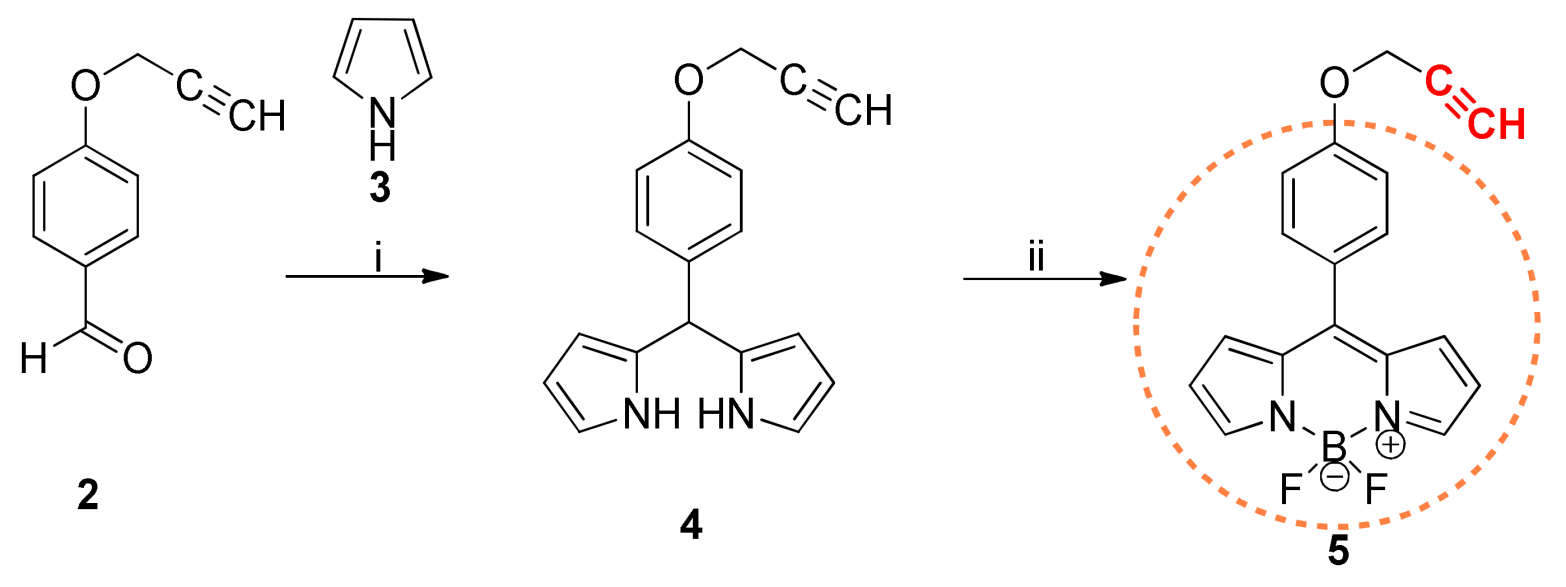
Reagents and conditions: (i) 0.1 equiv. TFA, excess of pyrrole, rt (room temperature), 10 min; (ii) 1 equiv. DDQ, CH_2_Cl_2_, 45 min, rt; 7 equiv. Et_3_N, 4 equiv. BF_3_·OEt_2_, rt, 3 h.

At first, we prepared the building elements for the couplings. That is, the BODIPY derivatives bearing terminal alkyne or azide functions (**5**, **11**) and the estrone derivatives possessing the complementary functions (**17**, **18**, **21**) were synthesized. BODIPY-alkyne **5** was synthesized using the aldehyde–pyrrole condensation strategy described above (Scheme 1). The propargylation of *p*-hydroxybenzaldehyde was efficiently achieved based on our earlier established procedure using propargyl bromide and potassium carbonate [31]. The dipyrrolylmethane core was built via trifluoroacetic acid (TFA) -catalyzed condensation of the aldehyde (**2**) and the pyrrole (**3**). The literature procedures were modified and combined in order to avoid the formation of oligomers [33,34]. Accordingly, a large excess of pyrrole was used, which served as both the reactant and the solvent in the condensation, allowing the selective formation of the dipyrrolylmethane. The desired compound **4** could efficiently be purified by flash chromatography by adding triethylamine to the eluent and covering the silica gel column with Al foil. Subsequent oxidation of the dipyrrolylmethane with DDQ (2,3-dichloro-5,6-dicyano-*p*-benzoquinone) and complexation with boron furnished the desired terminal alkyne (**5**) in high yield.

**Scheme 2:**
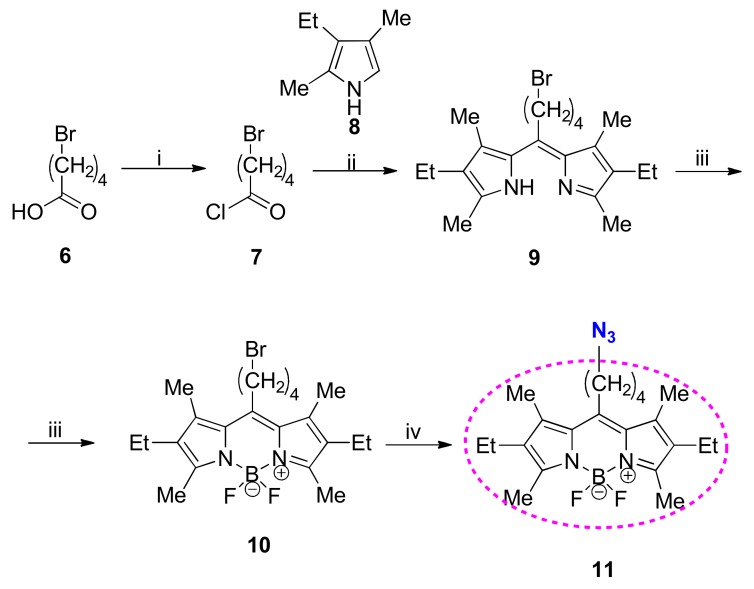
Reagents and conditions: (i) 1.5 equiv. oxalyl chloride, CH_2_Cl_2_, catalytic amount of *N*,*N*-dimethylformamide (DMF), rt, 2.5h; (ii) 2.5 equiv. of **8**, CH_2_Cl_2_, reflux, 4h; (iii) 20 equiv. Et_3_N, reflux, 30 min, 20 equiv. BF_3_·OEt_2_, reflux, 4 h; (iv) 1 equiv. of NaN_3_, DMSO, catalytic amount of AcOH.

The parent BODIPY derivative bearing azide function (**11**) was synthesized using the acyl chloride–pyrrole condensation strategy (Scheme 2) [35]. ω-Bromovaleric acid **6** was used as the azide precursor in order to build in a four-carbon-long spacer chain to the conjugate. Acyl chloride **7** was formed in situ with oxalyl chloride and a few drops of DMF. 3-Ethyl-2,4-dimethylpyrrole **8** was used for the condensation as a building block in order to avoid couplings at different positions or the formation of other side-products. Taking into consideration the different methodologies described in the literature [1,2,3,4,5,6,7,8,35], we tried to perform modifications in order to achieve the best yields and the highest chemoselectivity. We found that the order of reagent addition greatly influences the outcome of the condensation. It was found that the optimal conditions of the crucial step involved the use of diluted solutions of acyl chloride (**7**) and pyrrole (**8**). Additionally, the dropwise addition of the solution of **7** to that of **8** seemed to be superior to that of the reverse order. Subsequent complexation with BF_3_·OEt_2_ led to the bromo-BODIPY product (**10**) in high yield. The last step in the reaction sequence in the formation of the new azido-BODIPY product (**11**) was a bromine to azide exchange reaction using NaN_3_ as an azide source in the dimethyl sulfoxide (DMSO) solvent with a catalytic amount of AcOH.

**Scheme 3:**
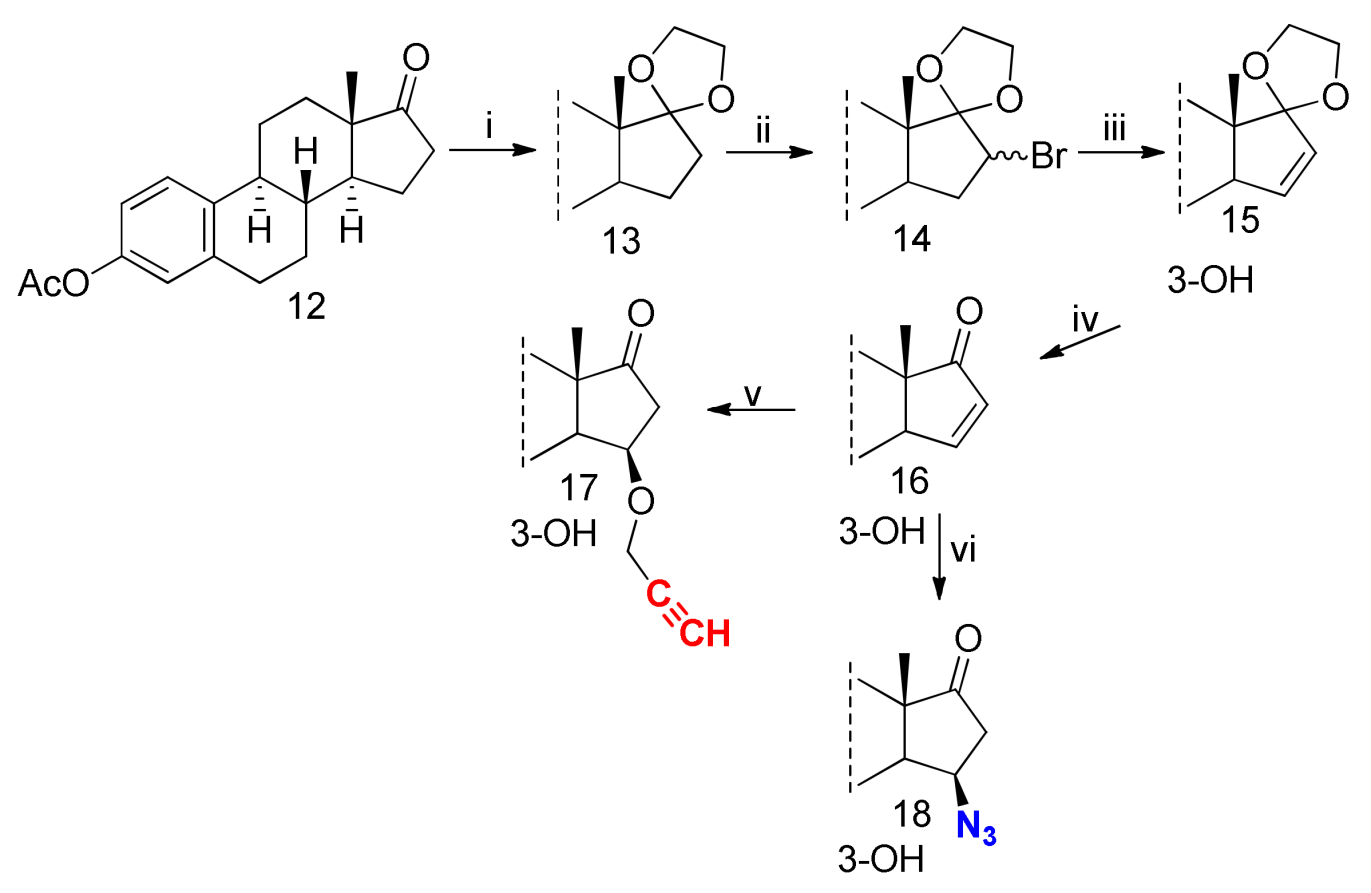
Reagents and conditions: (i) 2.5 equiv. of ethylene glycol; 2 equiv. of triethyl orthoformate; catalytic amount of *p*-TsOH; rt; 2 h; (ii) 1 equiv. of pyridinium hydrobromide perbromide; tetrahydrofurane (THF); rt; (iii) 2 equiv. of KO*^t^*Bu; DMSO; 80 °C; 1 h; (iv) 15 equiv. of H_2_CO; catalytic amount of *p*-TsOH; acetone; rt; 30 min; (v) propargyl alcohol (31 equiv.), catalytic amount of NaOH (5% aq), CH_2_Cl_2_, rt, 24 h; (vi) 1 equiv. of NaN_3_, DMSO, 4 equiv. of AcOH.

Next, we shifted our attention to the synthesis of estrone azide **18** and alkyne derivatives **17** and **21** suitable for CuAACs with BODIPY-alkyne **5** or -azide **11**. We earlier developed an efficient methodology for the generation of a Δ^15^-double bond in ring D of 3-*O*-methyl- and 3-*O*-benzyl-13α-estrone [36]. The α,β-unsaturated ketone **16** may serve as the key intermediate in the synthesis of 15-substituted estrone derivatives. Herein, we present the synthesis of the α,β-unsaturated ketone **16** in the 13β-estrone series without etherification of its 3-OH function (Scheme 3). We started with the 3-acetylated compound (**12**) developing a protecting group at C-17 in order to prevent the formation of undesired side-products during the reaction sequence. The next step was the reaction with pyridinium hydrobromide perbromide, which afforded the desired 16α-bromo derivative [37]. The dehydrobromination with potassium *tert*-butylate was accompanied by the hydrolysis of the acetate ester. The deprotection of the 17-ketal (**15**) led to the unsaturated ketone (**16**). The resulting ketone intermediate (**16**) was transformed into the appropriate alkyne (**17**) or azide (**18**) derivative by Michael addition. The nucleophile was stereoselectively introduced to C-15 in both cases, leading to the formation of one stereoisomer. The terminal alkyne function was introduced onto C-15 via *O*-propargylation with propargyl alcohol in dichloromethane, using a catalytic amount of NaOH as the base. The 15β-azide (**18**) was synthesized via the addition of HN_3_ formed in situ from NaN_3_ and AcOH. The data from the literature indicates 15β-orientation for the nucleophiles introduced via Michael addition onto the 3-protected α,β-unsaturated estrones [38]. We established the β-orientation of the functional group at C-15 in compound **17** through two-dimensional NMR measurements. The α-position of 15-H was deduced from the NOESY spectra since a cross-peak was observed between 15-H and 16α-H.

**Scheme 4:**
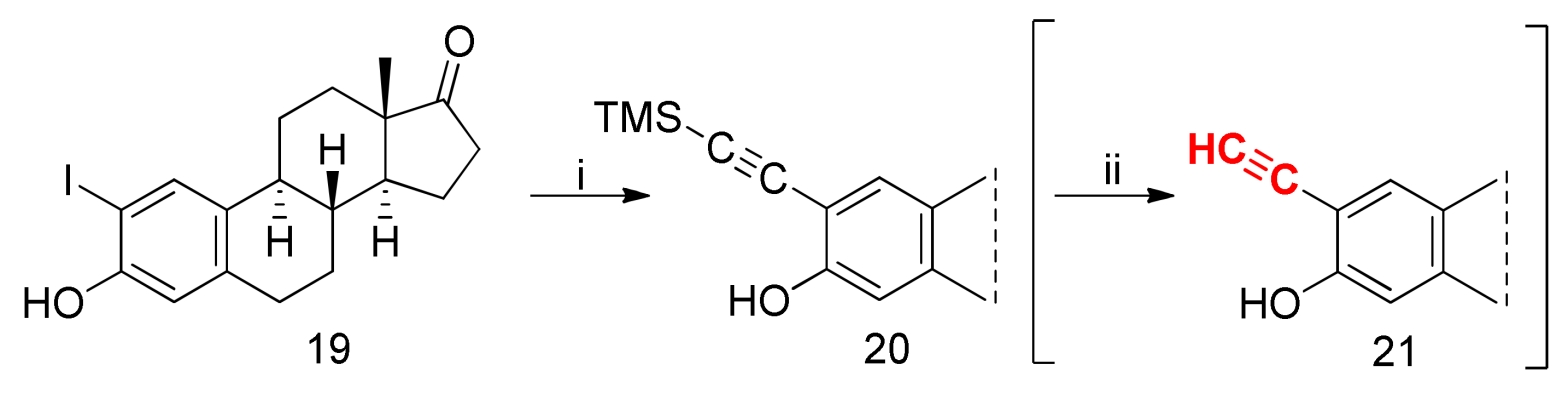
Reagents and conditions: (i) 0.1 equiv. of Pd(PPh_3_)_4_, 0.1 equiv. of CuI, 6 equiv. of Et_3_N, 2 equiv. of TMS-C≡CH, THF, MW, 50 °C, 20 min; (ii) 1 equiv. tetrabutylammonium fluoride (TBAF), toluene, 5 min.

The terminal alkyne function was introduced not only onto the C-15 but also directly onto the C-2 position via the Sonogashira reaction (Scheme 4). Coupling at C-2 could efficiently be achieved via our microwave-assisted Sonogashira procedure established earlier [39]. The synthesis of the steroidal 2-iodo derivative starting compound **19** has been reported by us recently [40]. It was reacted with trimethylsilylacetylene using 0.1 equiv. of Pd(PPh_3_)_4_ and 0.1 equiv. of CuI in tetrahydrofurane (THF) as a solvent in the presence of Et_3_N as a base at 50 °C for 20 min in a microwave reactor. The resulting 2-trimethylsilylethynyl-estrone (**20**) was purified by flash chromatography and served as a precursor of 2-ethynyl-estrone (**21**).

**Scheme 5:**
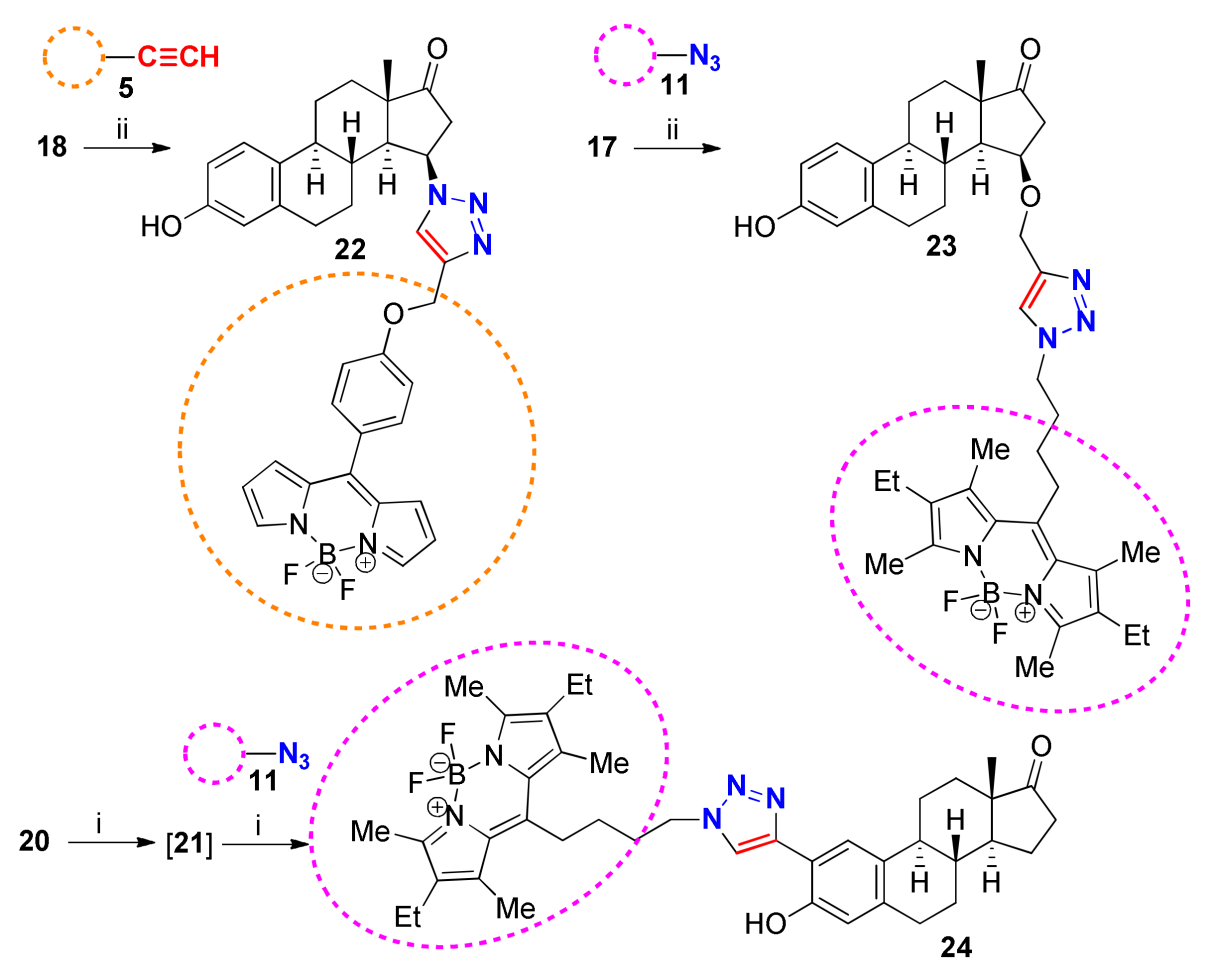
Reagents and conditions: (i) CuI (0.05 equiv.), Ph_3_P (0.1 equiv.), DIPEA (3 equiv.), 1 equiv. TBAF, toluene, reflux, 2 h; (ii) CuI (0.05 equiv.), Ph_3_P (0.1 equiv.), DIPEA (3 equiv.), toluene, reflux, 2 h.

With alkyne (**5**, **17**, **21**) and azide (**11**, **18**) complementers in hand, CuAAC reactions were carried out in order to synthesize the desired fluorescent estrone conjugates (Scheme 5). Estrone alkynes (**17**, **21**) or azides (**18**) were reacted with BODIPY-azide **11** or BODIPY-alkyne **5** under the previously reported CuAAC reaction conditions, using CuI as a catalyst, PPh_3_ as a ligand, and DIPEA as a base under conventional heating in toluene solvent. This protocol proved to be suitable for the efficient formation of the fluorescent conjugates (**22**–**24**). The 2-trimethylsilylethynyl-estrone derivative **20** underwent deprotection with tetrabutylammonium fluoride in situ during the click reaction. The conjugates (**22**–**24**) were regioselectively synthesized in high yields. The structures of the newly-synthesized labeled compounds (**22**–**24**) and their precursors were confirmed by ^1^H and ^13^C-NMR measurements.

**Figure 3:**
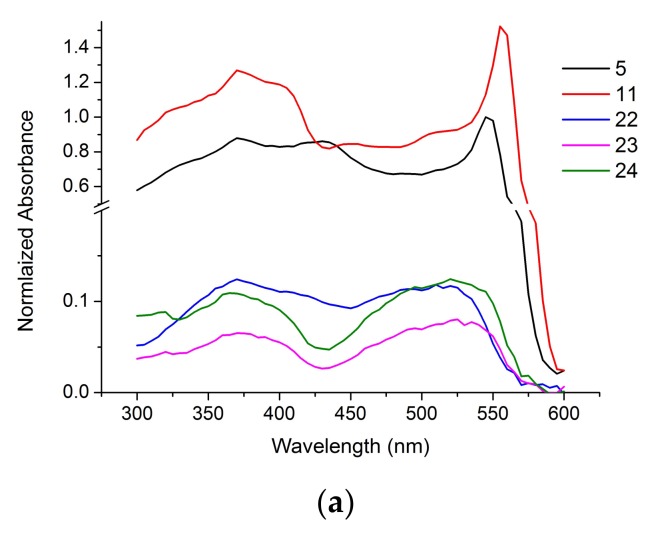

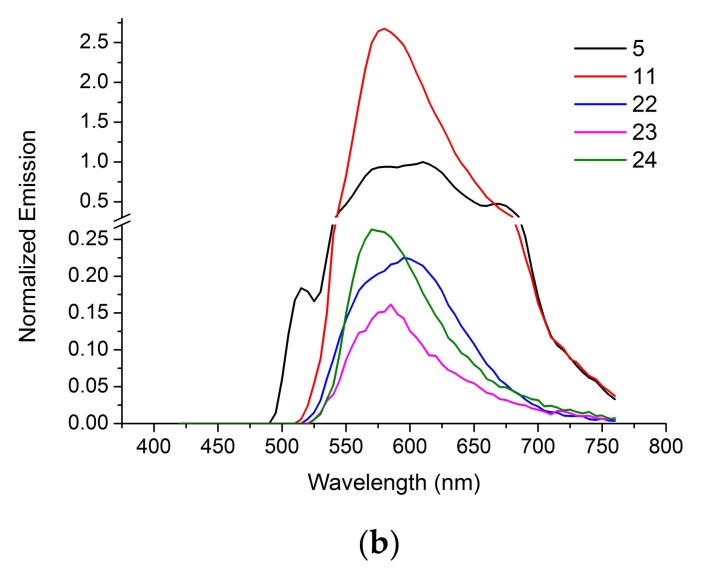
The normalized absorbance (**a**) and emission (**b**) spectra of the dyes (**5**, **11**) and the conjugates (**22**–**24**).

Figure 3 shows the absorption and the fluorescence emission spectra of the non-conjugated BODIPY dyes (**5**, **11**) and the steroid-BODIPY conjugates (**22**–**24**). Absorbance and emission spectra were normalized to maximum fluorescence of the unconjugated dye **5** measured at 545 nm emission and 610 nm excitation wavelengths, respectively. The absorption spectra of all the compounds have similar shape with two peaks of maximum absorption at 350–400 and 510–555 nm. The emission spectra of the dyes (**5**, **11**) and the conjugates (**22**–**24**) are also similar with a band around 580 nm for compounds **11, 23** and **24**; 600 nm for **22** and 610 nm for **5**. These data suggest the feasibility of the observation of conjugates **22**–**24** in living cells and tissues.

## 3. Materials and Methods

General

The melting points (m.p.) were determined with a Kofler hot-stage apparatus and are uncorrected. Elemental analyses were performed with a Perkin-Elmer CHN analyzer model 2400. Thin-layer chromatography: silica gel 60 F_254_; layer thickness 0.2 mm (Merck); eluent: a: 10% EtOAc/hexane, b: 2% EtOAc/CH_2_Cl_2_, and c: 20% EtOAc/CH_2_Cl_2_, if otherwise not stated, detection with I_2_ or UV (365 nm) after spraying with 5% phosphomolybdic acid in 50% aqueous phosphoric acid and heating at 100–120 °C for 10 min. Flash chromatography: silica gel 60, 40–63 μm (Merck). Reactions under microwave irradiation were carried out with a CEM Corporation focused microwave system, Model Discover SP. The ^1^H-NMR spectra were recorded in DMSO-d_6_, a CDCl_3_ solution with a Bruker DRX-500 instrument at 500 MHz, with Me_4_Si as an internal standard. The ^13^C-NMR spectra were recorded with the same instrument at 125 MHz under the same conditions. Mass spectrometry: a full scan mass spectra of the compounds were acquired in the range of 50–1000 *m*/*z* with a Finnigan TSQ-7000 triple quadrupole mass spectrometer (Finnigan-MAT, San Jose, CA, USA) equipped with a Finnigan electrospray ionization source. Analyses were performed in the positive ion mode using flow injection mass spectrometry with a mobile phase of 50% aqueous acetonitrile containing 0.1 *v*/*v*% formic acid. The flow rate was 0.3 mL/min. A 5-µL aliquot of the samples was loaded into the flow. The ESI capillary was adjusted to 4.5 kV and N_2_ was used as a nebulizer gas. Compound stocks prepared in DMSO (20 mM) were further diluted in 1× phosphate buffered saline, pH 7.4 to a final concentration of 500 µM. Absorption (at fixed 620 nm emission wavelengths) and emission (at fixed 400 nm excitation wavelength) spectra were measured using an Enspire plate reader (Perkin Elmer, Waltham, MA, USA).

### 3.1. Synthesis of BODIPY-alkyne ***5***

#### 3.1.1. Propargylation of 4-Hydroxybenzaldehyde

4-Hydroxybenzaldehyde (12.22 g, 10.0 mmol) was dissolved in acetone (100 mL) and then, propargyl bromide (1.7 mL (80 wt.% in toluene), 15.0 mmol) and K_2_CO_3_ (9.68 g, 70 mmol) were added. The reaction mixture was stirred at 70 °C for 24 h, the solvent was then evaporated off, and the residue was purified by flash chromatography with CH_2_Cl_2_/hexane = 80/20 as an eluent. Compound **2** was obtained as a white solid (14.9 g, 93%). Compound **2** is identical with that described in the literature [41,42].

#### 3.1.2. Synthesis of Dipyrrolylmethane **4**

To a solution of compound **2** (0.48 g, 3 mmol) and pyrrole (9.0 mL, 1.29 mol), trifluoroacetic acid (0.024 mL, 0.3 mmol) was added. The solution was stirred for 10 min at room temperature under a nitrogen atmosphere. The mixture was diluted with CH_2_Cl_2_ (200 mL), washed with 0.1 M aq NaOH, water, and dried Na_2_SO_4_. The solvent and a great amount of unreacted pyrrole were removed under reduced pressure. The residue was purified by flash chromatography on a silica gel column covered with Al foil with hexane/EtOAc/Et_3_N = 80/20/1 as an eluent. Compound **4** was obtained as a brown solid (550 mg, 67%). Compound **4** is identical with that described in the literature [41,42].

#### 3.1.3. Synthesis of Boron Dipyrrolylmethene **5**

Dipyrrolylmethane (**4**, 0.6 g, 2.2 mmol) was dissolved in dry CH_2_Cl_2_ (30 mL), then DDQ (0.49 g, 2.2 mmol) was added and the reaction mixture was stirred for 1 h at rt (room temperature) under a nitrogen atmosphere. This was followed by the successive addition of Et_3_N (2 mL, 14.4 mmol) and BF_3_·Et_2_O (2.3 mL, 8.6 mmol) and the stirring was continued for 4 h. The reaction was quenched by the addition of a saturated NaHCO_3_ solution (25 mL). The organic layer was separated and dried over with Na_2_SO_4_. The solvents were removed under reduced pressure and the residue was purified by flash chromatography with EtOAc/hexane = 20/80 as an eluent. Compound **5** was obtained as a tawny solid (550 mg, 78%). M.p.: 147–150 °C, R_f_ = 0.78^b^. Compound **5** is identical with that described in the literature [34]. ^1^H-NMR (CDCl_3_) δ (ppm): 2.60 (s, 1H, C≡CH); 4.80 (s, 2H, OCH_2_); 6.55 (s, 2H); 6.97 (s, 2H); 7.14 (m, 2H); 7.56 (m, 2H); 7.92 (s, 2H).

### 3.2. Synthesis of BODIPY-azide ***11***

#### 3.2.1. Synthesis of BODIPY-bromide **10**

DMF (3 drops) and oxalyl chloride (0.5 mL, 5.8 mmol) were added to a solution of 5-bromovaleric acid (**6**, 0.76 g, 4.2 mmol) in dry CH_2_Cl_2_ (20 mL) and the mixture was stirred for 3 h at rt. Then the mixture was evaporated with toluene (3 × 10 mL). The residue was re-dissolved in anhydrous CH_2_Cl_2_ (20 mL) and the resulting solution was added dropwise to a stirred solution of 3-ethyl-2,4-dimethylpyrrole (**8**, 1.23 g, 10 mmol) in dry dichloromethane (20 mL). After heating under reflux for 3 h, the mixture was cooled to rt and triethylamine (11 mL, 80 mmol) was added. After a 30 min reflux, BF_3_·Et_2_O (10 mL, 80 mmol) was added and the reaction mixture was stirred for 3 h under reflux. After cooling to rt, the reaction was quenched by the addition of a saturated NaHCO_3_ solution (30 mL) and extracted with dichloromethane (3 × 20 mL). The combined organic layers were dried (Na_2_SO_4_) and the solvents were removed under a reduced pressure. The residue was purified by flash chromatography with EtOAc/hexane = 10/90 as an eluent. Compound **10** was obtained as a red solid (1.01 g, 55%). M.p.: 160–161 °C, R_f_ = 0.54^a^. Compound **10** is identical with that described in the literature without characterization [43]. Anal calcd. for C_21_H_30_BBrF_2_N_2_: C, 57.43; H, 6.89. Found: C, 57.52; H, 6.73. ^1^H-NMR (DMSO-d_6_) δ (ppm): 1.05 (t, 6H, *J* = 7.5 Hz, 2xCH_2_CH_3_); 1.80 (m, 2H), 2.06 (m, 2H); 2.34 and 2.50 (2xs, 2x6H, 4xCH_3_); 2.40 (q, 4H, *J* = 7.5 Hz, 2xCH_2_CH_3_); 3.02 (m, 2H); 3.45 (m, 2H). ^13^C-NMR δ (ppm): 12.4 (2C); 13.4 (2C), 14.8 (2C); 17.2 (2C); 27.6; 30.2; 32.7; 33.1; 130.9; 132.7 (2C); 135.5 (2C), 143.5 (2C); 152.4 (2C). MS *m*/*z* (%): 419 (100, [M − F]^+^).

#### 3.2.2. Synthesis of BODIPY-azide **11**

A stirred solution of compound **10** (878 mg, 2.0 mmol) in dry DMSO (5 mL) was treated with acetic acid (3 drops) and NaN_3_ (130 mg, 2.0 mmol). The mixture was stirred for 0.5 h at rt, poured into water (50 mL), and extracted with dichloromethane (3 × 20 mL). The combined organic layers were dried (Na_2_SO_4_) and the solvents were removed under a reduced pressure. The residue was purified by flash chromatography with EtOAc/hexane = 10/90 as an eluent. Compound **11** was obtained as a red solid (746 mg, 92%). M.p.: 110–112 °C, R_f_ = 0.50^a^. Anal calcd. for C_21_H_30_BF_2_N_5_: C, 62.85; H, 7.53. Found: C, 62.92; H, 7.47. ^1^H-NMR (DMSO-d_6_) δ (ppm): 1.05 (t, 6H, *J* = 7.5 Hz, 2xCH_2_CH_3_); 1.73–1.80 (overlapping multiplets, 4H); 2.33 and 2.50 (2xs, 2x6H, 4xCH_3_); 2.41 (q, 4H, *J* = 7.5 Hz, 2xCH_2_CH_3_); 3.02 (m, 2H); 3.37 (m, 2H). ^13^C-NMR δ (ppm): 12.4 (2C); 13.3 (2C), 14.8 (2C); 17.2 (2C); 27.8; 28.8; 29.2; 51.0; 130.9; 132.7 (2C); 135.5 (2C), 143.6 (2C); 152.4 (2C). MS *m*/*z* (%): 382 (100, [M − F]^+^).

### 3.3. Synthesis of α,β-Unsaturated Ketone Intermediate ***16***

#### 3.3.1. Synthesis of 16-Bromo-17-ketal **14**

To a suspension of **12** (13.75 g, 44 mmol) in triethyl orthoformate (15 mL, 88 mmol) and ethylene glycol (6 mL, 110 mmol), a catalytic amount of *p*-TsOH was added, and the mixture was gently heated for 0.5 h until the suspension transformed into a clear solution. The warm reaction mixture was then poured into a saturated solution of sodium bicarbonate (100 mL). The white precipitate formed was filtered off, washed with water, and dried in air. To a solution of crude product **13** (10.70 g, 30 mmol) in dry tetrahydrofuran (100 mL), 10 g (30 mmol) of pyridinium hydrobromide perbromide was added. A white precipitate formed immediately from the yellow solution. The reaction mixture was stirred at rt for 2 h, then it was diluted with a saturated aqueous solution of sodium bicarbonate (500 mL). The precipitate formed was filtered off, washed with water, and dried in air. The crude product reacted further without purification. Compound **14** is identical with that described in Reference [44].

#### 3.3.2. Synthesis of α,β-Unsaturated Ketal **15**

To a solution of **14** (4.35 g, 10 mmol) in dimethyl sulfoxide (50 mL), potassium *tert*-butylate (2.24 g, 20 mmol) was added. The suspension was vigorously stirred and heated (80 °C) for 5 h until it became a clear yellow-brown solution. The mixture was poured into ice-water (1 L) and the resulting precipitate was filtered off, washed with water, and dried in air. The crude product was subjected to chromatographic separation with dichloromethane as an eluent. Compound **15** was obtained as a white solid (2.6 g, 83%). Compound **15** is identical with that described in Reference [44].

#### 3.3.3. Synthesis of α,β-Unsaturated Ketone **16**

To a solution of **15** (1.25 g, 4 mmol) in acetone (25 mL), formaldehyde (5 mL, 37% in water, 60 mmol formaldehyde) and a catalytic amount of *p*-TsOH were added. The mixture was stirred for 1.5 h at rt and then poured into water (100 mL). The precipitate formed was filtered off, washed with water, and dried in air. The crude product (**16**) was subjected to chromatographic separation with EtOAc/CH_2_Cl_2_ = 2/98 as an eluent. Compound **16** was obtained as a white solid (1.0 g, 84%). M.p.: 245–246 °C, R_f_ = 0.16^b^. Compound **16** is identical with that discussed in the literature without full characterization [44]. Anal calcd. for C_18_H_20_O_2_: C, 80.56; H, 7.51. Found: C, 80.64; H, 7.42. ^1^H-NMR (DMSO-d_6_) δ (ppm): 0.99 (s, 3H, 18-H_3_); 2.79 (m, 2H, 6-H_2_); 6.05 (d, 1H, *J* = 2.5 Hz, 16-H); 6.47 (d, 1H, *J* = 2.2 Hz, 4-H); 6.52 (double doublet 1H, *J* = 8.6 Hz, *J* = 2.2 Hz, 2-H); 7.04 (d, 1H, *J* = 8.6 Hz, 1-H); 7.84 (d, 1H, *J* = 5.6 Hz, 15-H); 9,02 (s, 1H, OH). ^13^C-NMR δ (ppm): 20.5 (C-18); 25.0; 26.0; 28.6; 28.8; 35.2; 44.3; 50.7; 55.2; 112.7; 114.9; 125.6; 129.8; 130.8; 136.9; 155.0; 159.5; 211.9. MS *m*/*z* (%): 310 (100); 269 (66, [M + H]^+^).

### 3.4. Synthesis of Steroidal Alkyne ***17***

To a stirred solution of compound **16** (268.4 mg, 1.0 mmol) in dry CH_2_Cl_2_ (15 mL), propargyl alcohol (3.6 mL, 31 mmol) and 5 drops of 5% aq. NaOH were added. The reaction mixture was stirred at rt for 24 h. The reaction was quenched by the addition of water (150 mL) and extracted with dichloromethane (3 × 20 mL). The combined organic layers were dried (Na_2_SO_4_) and the solvent was removed under a reduced pressure. The residue was purified by flash chromatography with CH_2_Cl_2_ as an eluent. Compound **17** was obtained as a white solid (256 mg, 79%). M.p.: 167–169 °C, R_f_ = 0.20^b^. Anal calcd. for C_21_H_24_O_3_: C, 77.75; H, 7.46. Found: C, 77.83; H, 7.38. ^1^H-NMR (DMSO-d_6_) δ (ppm): 1.03 (s, 3H, 18-H_3_); 2.75 (m, 2H, 6-H_2_); 3.44 (s, 1H, C≡CH); 4.11 and 4.25 (2xm, 2x1H, OCH_2_); 4.37 (t, 1H, *J* = 5.1 Hz, 15-H); 6.46 (d, 1H, *J* = 2.2 Hz, 4-H); 6.51 (d, 1H, *J* = 8.6 Hz, *J* = 2.2 Hz, 2-H); 7.05 (d, 1H, *J* = 8.6 Hz, 2-H); 9.03 (s, 1H, OH). ^13^C-NMR δ (ppm): 17.2 (C-18); 25.3 (2C); 28.9; 32.3; 34.6; 42.3; 43.6; 46.5; 52.9; 55.6 (OCH_2_); 73.1 (C-15); 76.9 (C≡CH); 80.4 (C≡CH); 112.7 (C-2); 114.9 (C-4); 125.8 (C-1); 129.9 (C-10); 137.0 (C-5); 154.9 (C-3); 218.6 (C-17).

### 3.5. Synthesis of the Steroidal Azide ***18***

A stirred solution of compound **16** (268 mg, 1.0 mmol) in dry DMSO (5 mL) was treated with acetic acid (0.24 mL, 4.0 mmol) and NaN_3_ (65 mg, 1.0 mmol). The mixture was stirred for 0.5 h at rt, poured into water (50 mL), and extracted with dichloromethane (3 × 20 mL). The combined organic layers were dried (Na_2_SO_4_) and the solvents were removed under a reduced pressure. The residue was purified by flash chromatography with EtOAc/hexane = 1/99 as an eluent. Compound **18** was obtained as a white solid (252 mg, 81%). M.p.: 138–139 °C, R_f_ = 0.24^b^. Anal calcd. for C_18_H_21_N_3_O_2_: C, 69.43; H, 6.80. Found: C, 69.48; H, 6.72. ^1^H-NMR (DMSO-d_6_) δ (ppm): 0.89 (s, 3H, 18-H_3_); 2.76 (m, 2H, 6-H_2_); 4.16 (m, 1H, 15-H); 6.44 (d, 1H, *J* = 2.2 Hz, 4-H); 6.52 (dd, *J* = 8.6 Hz*, J* = 2.2 Hz, 2-H); 7.05 (d, 1H, *J* = 8.6 Hz, 1-H); 9.04 (s, 1H, OH). ^13^C-NMR δ (ppm): 14.7 (C-18); 25.6; 26.3; 29.0; 31.0; 37.7; 42.0; 43.1; 49.7; 53.1; 58.1; 112.8; 114.7; 126.3; 129.3; 136.8; 155.0; 214.4.

### 3.6. Synthesis of Triazoles ***22*** and ***23***

To a stirred solution of **5** (322 mg, 1.0 mmol) or **17** (324 mg, 1.0 mmol) in toluene (5 mL), Ph_3_P (26 mg, 0.1 mmol), CuI (9.5 mg, 0.05 mmol), DIPEA (0.52 mL, 3.0 mmol), and **18** (311 mg, 1.0 mmol) or **11** (401 mg, 1.0 mmol) were added. The reaction mixture was kept at the reflux temperature for 2 h and then allowed to cool and evaporate in vacuo. The residue was purified by flash chromatography with EtOAc/CH_2_Cl_2_ = 10/90 as an eluent.

Compound **22** was obtained as a red solid (557 mg, 88%). M.p.: 273–275 °C, R_f_ = 0.42^c^. Anal calcd. for C_36_H_34_BF_2_N_5_O_3_: C, 68.55; H, 5.41. Found: C, 68.63; H, 5.33. ^1^H-NMR (DMSO-d_6_) δ (ppm): 1.03 (s, 3H, 18-H_3_); 5.34 (s, 2H, OCH_2_); 5.42 (m, 1H, 15-H); 6.32 (d, 1H, *J* = 2.2 Hz, 4-H); 6.50 (dd, 1H, *J* = 8.6 Hz, *J* = 2.2 Hz, 2-H); 6.64 (m, 2H); 7,01 (m, 2H); 7,03 (d, 1H, *J* = 8.6 Hz, 1-H); 7.27 (m, 2H); 7.64 (m, 2H); 8.09 (s, 2H); 8.59 (s, 1H); 9.00 (s, 1H). ^13^C-NMR δ (ppm): 14.5 (C-18); 24.4; 28.6; 31.0; 37.7; 43.0; 44.0; 50.0; 53.2; 57.6; 61.4; 112.8; 114.6; 115.1 (2C); 118.9 (2C); 123.4; 125.6; 126.2; 129.1; 131.4 (2C); 132.5 (2C); 133.9; 136.5; 142.8; 143.9 (2C); 146.8; 154.9; 160.5; 213.6. MS *m*/*z* (%): 143 (100); 634 (80, [M + H]^+^).

Compound **23** was obtained as a red solid (588 mg, 81%). M.p.: 177–179 °C, R_f_ = 0.24^c^. Anal calcd. for C_42_H_54_BF_2_N_5_O_3_: C, 69.51; H, 7.50. Found: C, 69.59; H, 7.40. ^1^H-NMR (DMSO-d_6_) δ (ppm): 0.95 (t, 6H, *J* = 7.5 Hz, 2xCH_2_CH_3_); 1.01 (s, 3H, 18-H_3_); 2.24 (overlapping multiplets, 4H, 2xCH_2_CH_3_); 2.33 (overlapping singlets, 12H, 4xCH_3_); 2.96 (m, 2H, 6-H_2_); 4.13 (m, 1H, 15-H); 4.41 and 4.61 (2xd, 2x1H, *J* = 12.5 Hz, OCH_2_); 4.45 (m, 2H); 6.39 (d, 1H, *J* = 2.2 Hz, 4-H); 6.50 (dd, 1H, *J* = 8.6 Hz, *J* = 2.2 Hz, 2-H); 7.01 (d, 1H, *J* = 8.6 Hz, 1-H); 8.10 (s, 1H, C≡CH); 9.01 (s, 1H, OH). ^13^C-NMR δ (ppm): 12.7 (2C); 14.0 (2C); 14.6 (2C); 16.4; 17.2 (C-18); 25.3; 25.4; 27.2; 28.0; 28.8; 30.0; 32.3; 34.4; 42.6; 43.3; 46.5; 48.9; 53.0; 54.8; 61.6; 73.0; 112.5; 114.9; 123.9; 125.7; 130.0 (3C); 132.2 (2C); 136.0; 136.1; 137.0; 143.9 (2C); 144.3; 154.9 (2C); 218.7. MS *m*/*z* (%): 706 (100, [M − F]^+^).

### 3.7. Synthesis of 3-Hydroxy-2-trimethylsilylethynyl-estra-1,3,5(10)-trien-17-one ***20***

3-Hydroxy-2-iodo-estra-1,3,5(10)-trien-17-one (**19**, 276 mg, 1.0 mmol), Pd(PPh_3_)_4_ (115 mg, 0.01 mmol), CuI (19 mg, 0.01 mmol), and THF (3 mL) were added together under a nitrogen atmosphere. Then, Et_3_N (0.56 mL, 4.0 mmol) was added and the mixture was stirred at 50 °C for 10 min. Ethynyltrimethylsilane (0.28 mL, 2.0 mmol) was added with a syringe into 10 mL Pyrex pressure vessels (CEM, Part #: 908035) with silicone caps (CEM, Part #: 909210) and the mixture was heated in a CEM microwave reactor at 50 °C for 20 min under stirring. The solvent was evaporated in vacuo and the residue was purified by flash chromatography with diisopropyl ether/hexane = 50/50 as an eluent. Compound **20** was obtained as a white solid (1.0 g, 84%). M.p.: 179–181 °C, R_f_ = 0.26^a^. Anal calcd. for C_23_H_30_O_2_Si: C, 75.36; H, 8.25. Found: C, 75.42; H, 8.16 ^1^H-NMR (CDCl_3_) δ (ppm): 0.26 (s, 9H, 3xSi-CH_3_); 2.87 (m, 2H, 6-H_2_); 5.62 (s, 1H, OH); 6.67 (s, 1H, 4-H); 7.25 (s, 1H, 1-H). ^13^C-NMR δ (ppm): 0.01 (3C, 3xSi-CH_3_); 13.8 (C-18); 21.6; 25.8; 26.3; 29.5; 31.4; 35.8; 38.1; 43.7; 47.9 (C-13); 50.4; 99.4; 101.3; 106.9, 114.3; 128.5; 131.9; 139.9; 154.8 (C-3); 220.8 (C-17)

### 3.8. Synthesis of BODIPY–estrone Conjugate ***24***

To a stirred solution of **20** (367 mg, 1.0 mmol) in toluene (5 mL), Ph_3_P (26 mg, 0.1 mmol), CuI (9.5 mg, 0.05 mmol), DIPEA (0.52 mL, 3.0 mmol), and **11** (1 equiv.) were added. The reaction mixture was treated at boiling temperature for 0.5 h and TBAF (1 mL, 1 mmol) was added. The heating was continued for 2 h at the reflux temperature. The mixture was allowed to cool and evaporate in vacuo. The residue was purified by flash chromatography with EtOAc/CH_2_Cl_2_ = 10/90 as an eluent. Compound **24** was obtained as a red solid (550 mg, 79%). M.p.: 218–220 °C, R_f_ = 0.26^b^. Anal calcd. for C_41_H_52_BF_2_N_5_O_2_: C, 70.78; H, 7.53. Found: C, 70.85; H, 7.41. ^1^H-NMR (CDCl_3_) δ (ppm): 0.95 (s, 3H, 18-H_3_); 1.01 (t, 6H, *J* = 7.5 Hz, 2xCH_2_CH_3_); 2.26 and 2.47 (2xs, 2x6H, 4xCH_3_); 2.37 (q, 4H, *J* = 7.5 Hz, 2xCH_2_CH_3_); 2.90 (m, 2H, 6-H_2_); 3.05 (m, 2H); 4.46(m, 2H, N-CH_2_); 6.79 (s, 1H, 4-H); 7.29 (s, 1H, 1-H); 7.79 (s, 1H, C≡CH/OH). ^13^C-NMR δ (ppm): 12.4 (2C); 13.4 (2C); 13.8 (2C); 14.8 (C-18); 17.1; 21.6; 26.0; 26.4; 27.5; 28.6; 29.2; 30.3; 31.5; 35.9; 38.2; 43.8; 48.0; 50.4; 50.5; 111.5; 117.3; 118.6; 122.6; 130.8 (2C); 131.1; 132.9 (2C); 135.3 (2C); 138.7; 142.9 (2C); 147.9; 152.6; 153.7; 220.9(C-17) MS *m*/*z* (%): 696 (100, [M + H]^+^). MS *m*/*z* (%): 696 (100, [M + H]^+^).

## 4. Conclusions

In conclusion, we described here a facile and efficient synthetic route to C-2- or C-15-labeled BODIPY–estrone conjugates via Sonogashira and/or click chemistry. Our strategy enables variations in the length of linkers, thereby providing a library of fluorescing conjugates. The selection of the site of conjugation as well as the nature of the substituents on the estrone moiety and the use of different linkers allow for the determination of the effect of structural modifications on the biological properties of the labeled compound. The methodologies developed should find extensive applications owing to the great importance of fluorescent labeled biomolecules. The newly synthesized labeled estrone derivatives may serve as good candidates for the development of imaging probes for biological assays.

## Supplementary Materials

Supplementary File 1
