# Supplementary material for: Synthesis of Novel C-2- or C-15-Labeled BODIPY—Estrone Conjugates
Source: Molecules. 2018 Apr 3;23(4):821. doi: 10.3390/molecules23040821 (PMC6017578; doi:10.3390/molecules23040821)
Supplement: Supplementary File 1 [file molecules-23-00821-s001.pdf]

Supporting Information for "Synthesis of novel C-2- or C-15-labeled BODIPY-estrone conjugates"

Ildikó Bacsa [1], Csilla Konc [1], Anna Boglárka Orosz [1], Gábor Kecskeméti [2], Réka Rigó [3], Csilla Özvegy-Laczka [3] and Erzsébet Mernyák [1,*]

[1] Department of Organic Chemistry, University of Szeged, Dóm tér 8, H-6720 Szeged, Hungary;

[2] Department of Medicinal Chemistry, University of Szeged, Dóm tér 8, H-6720 Szeged, Hungary;

[3] Membrane protein research group, Institute of Enzymology, Research Centre for Natural Sciences, Hungarian Academy of Sciences, Magyar tudósok körútja 2, H-1117 Budapest, Hungary

* Correspondence: bobe@chem.u-szeged.hu; Tel.: +36 62 544277

NMR spectras

11

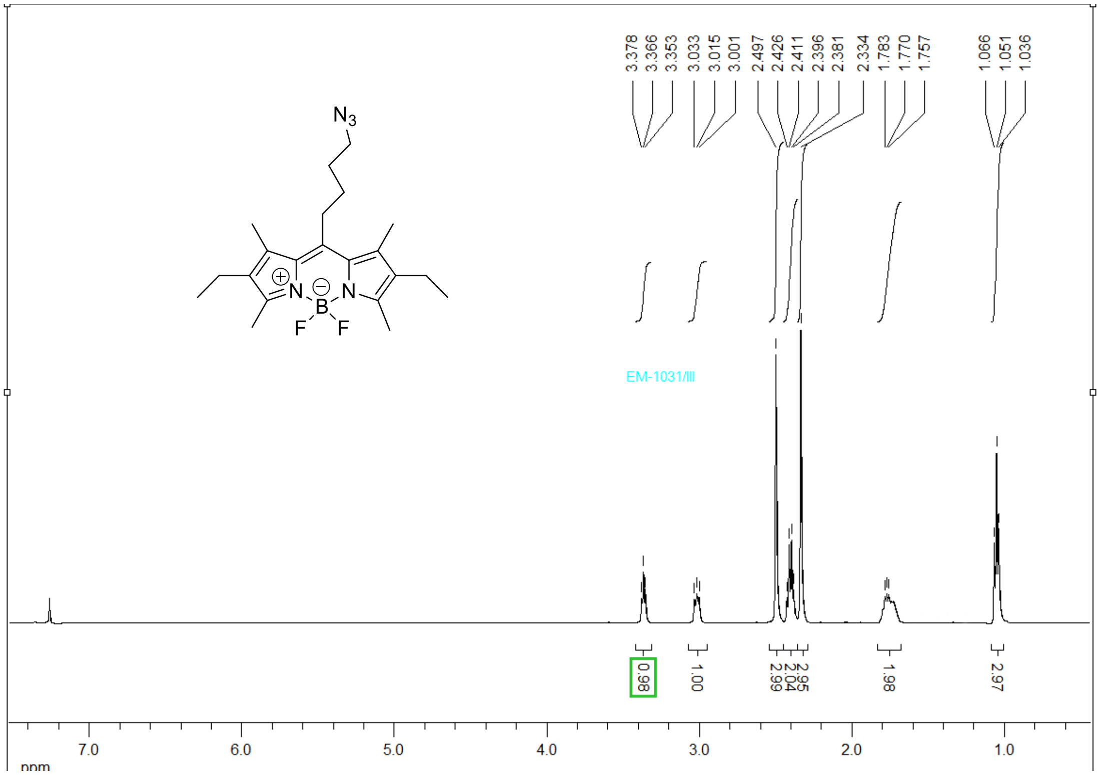

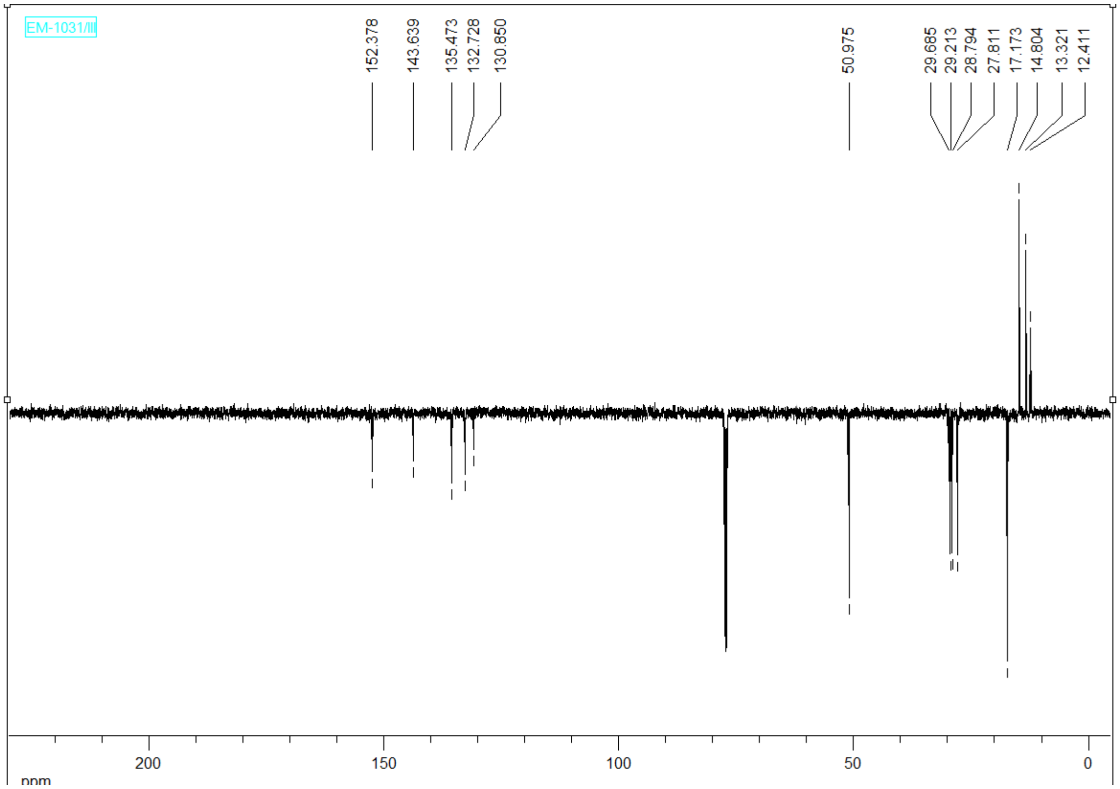

16

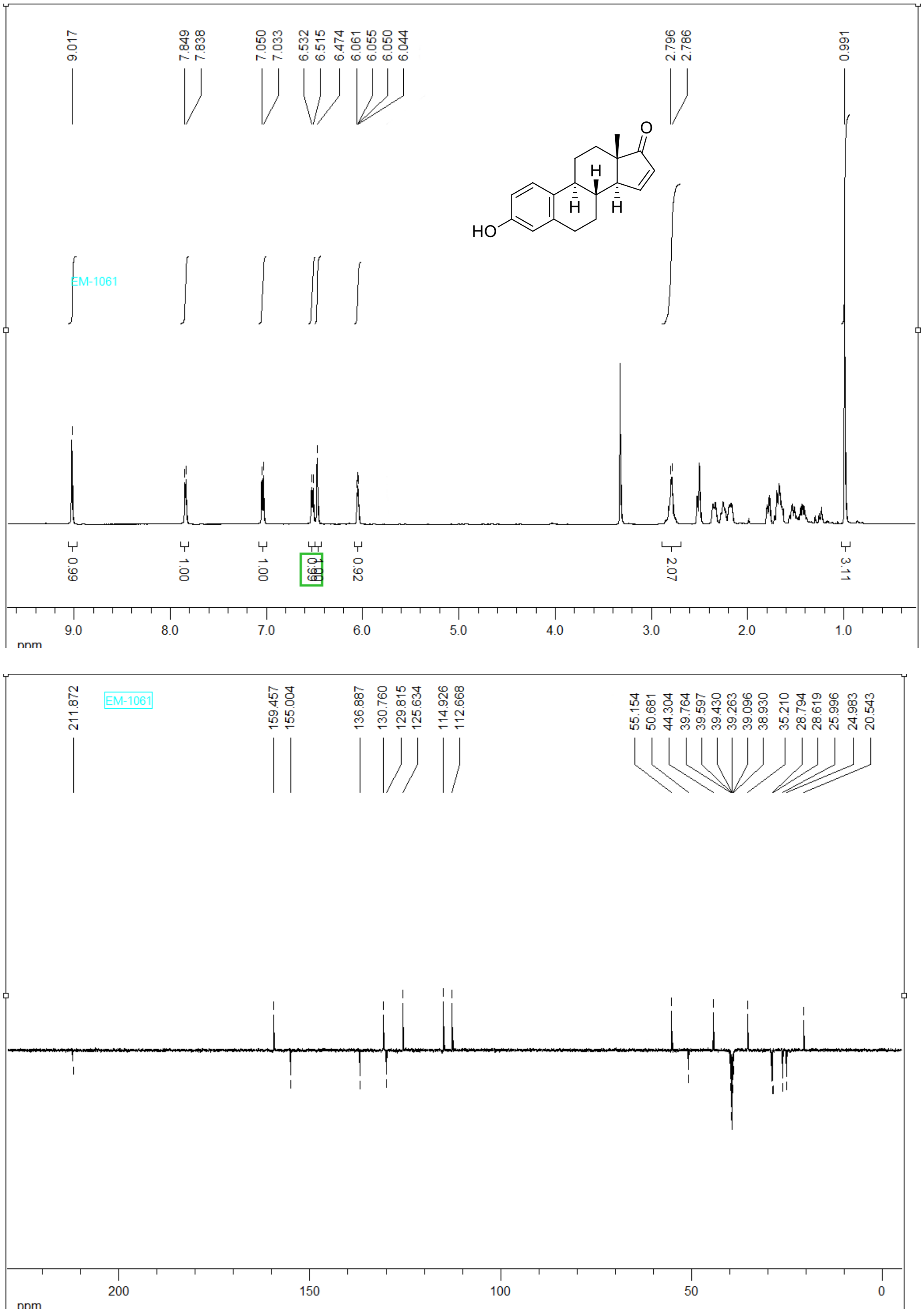

9.017
7.849
7.838
7.050
7.033
6.532
6.515
6.474
6.061
6.055
6.050
6.044
2.796
2.786
0.991
EM-1061
HO
H
O
0.99
1.00
1.00
0.92
2.07
3.11
9.0
8.0
7.0
6.0
5.0
4.0
3.0
2.0
1.0
ppm
211.872
EM-1061
159.457
155.004
136.887
130.760
129.815
125.634
114.926
112.668
55.154
50.681
44.304
39.764
39.597
39.430
39.263
39.096
38.930
35.210
28.794
28.619
25.996
24.983
20.543
200
150
100
50
0
ppm

17

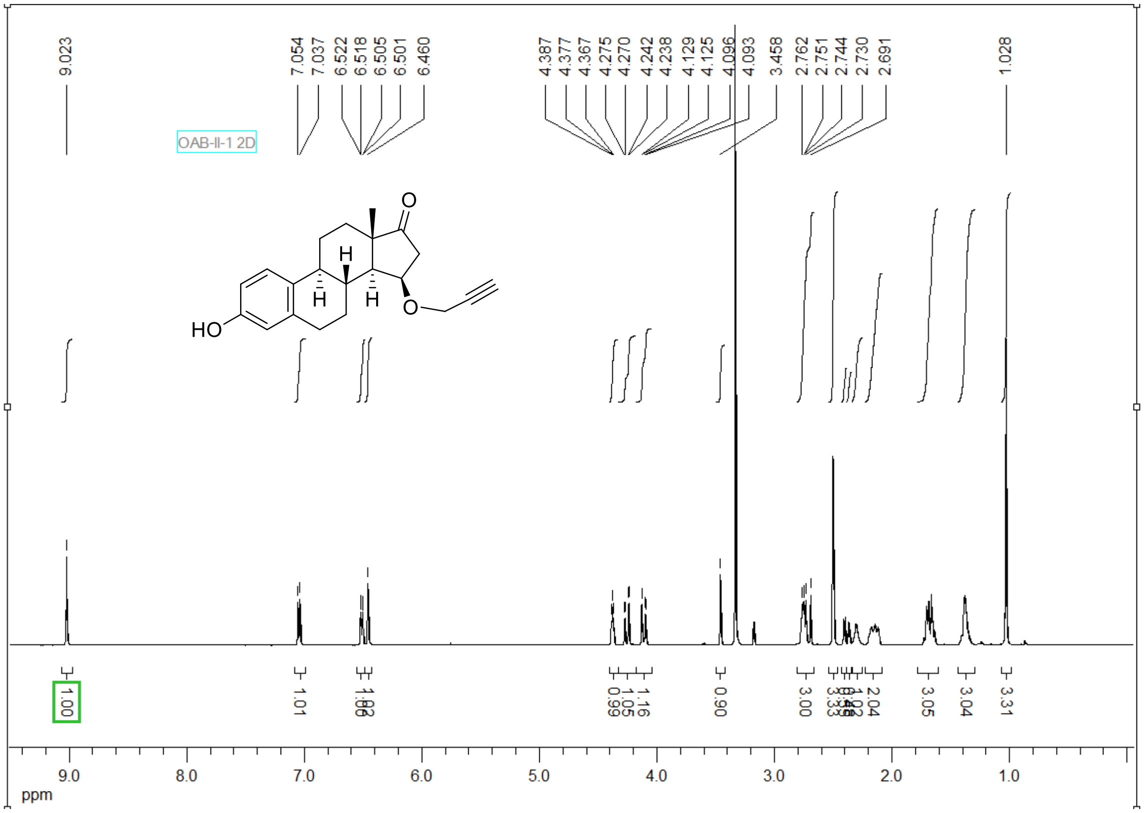

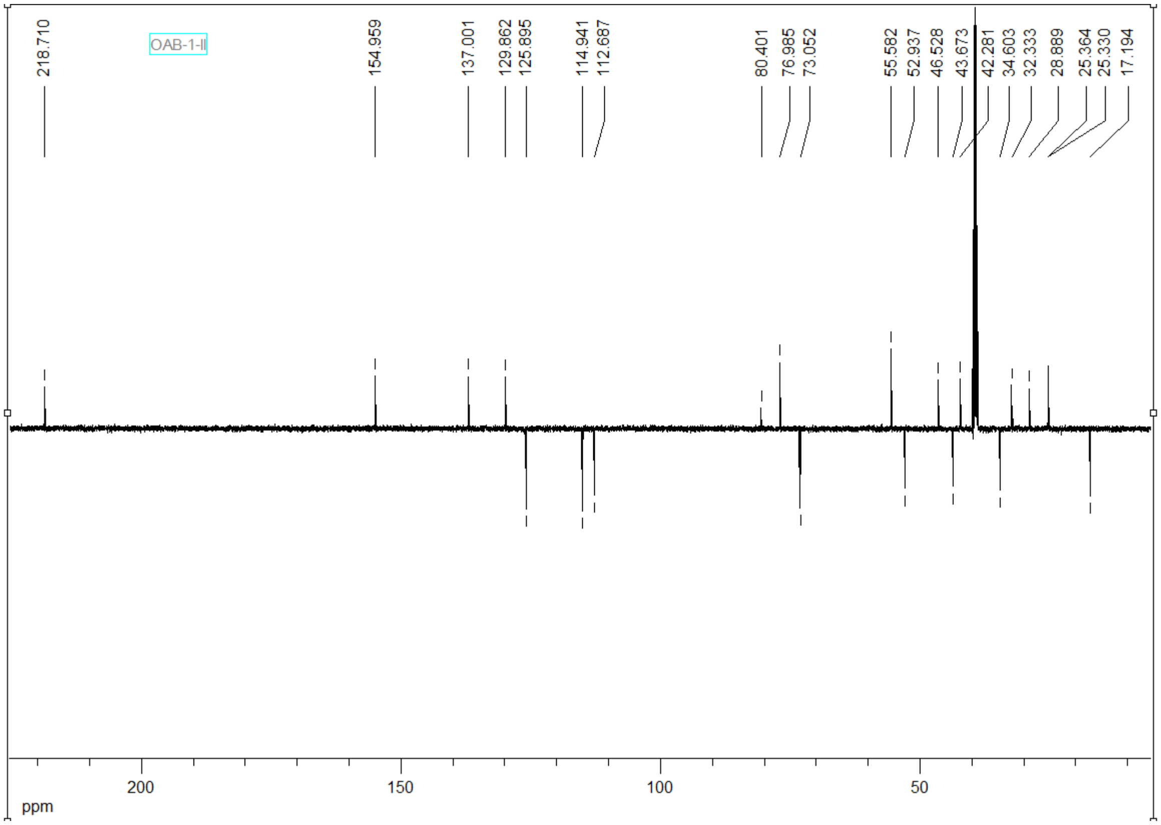

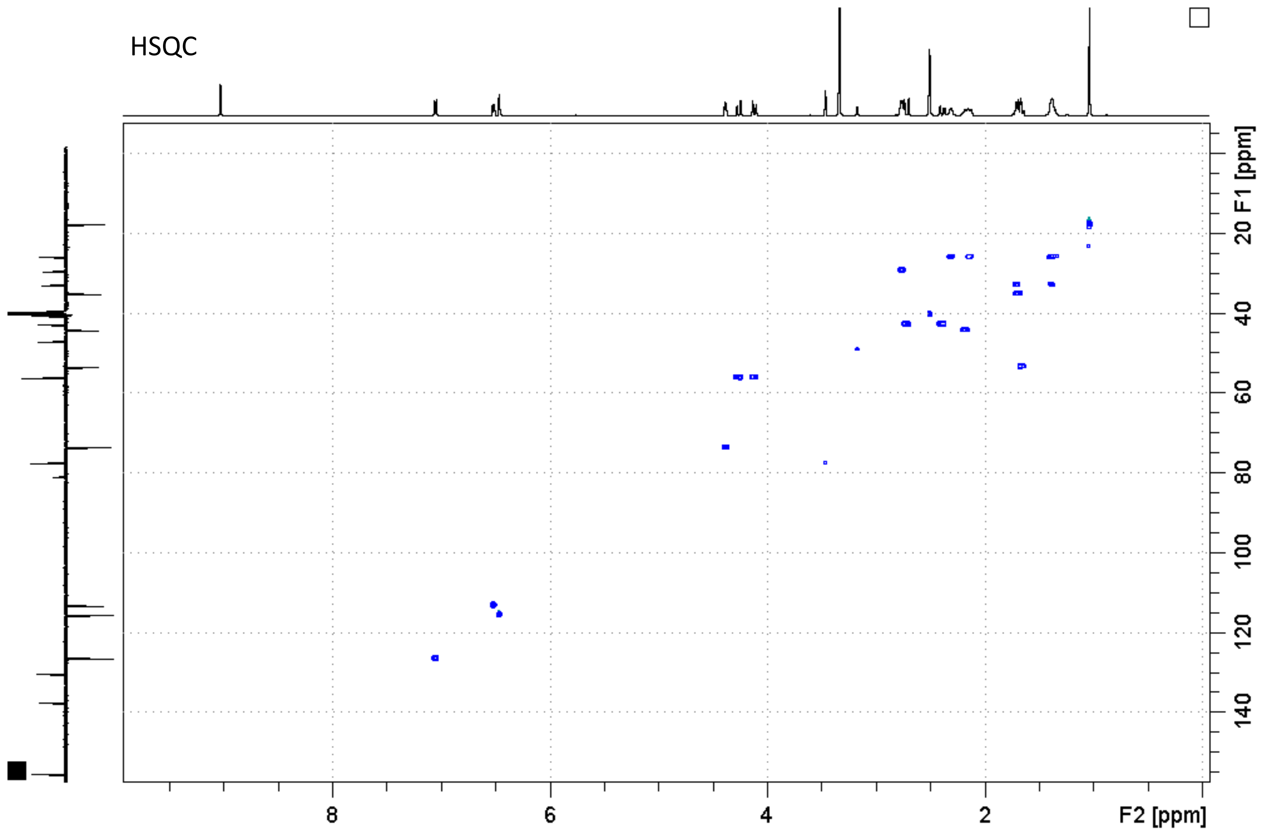
HSQC
F1 [ppm]
F2 [ppm]

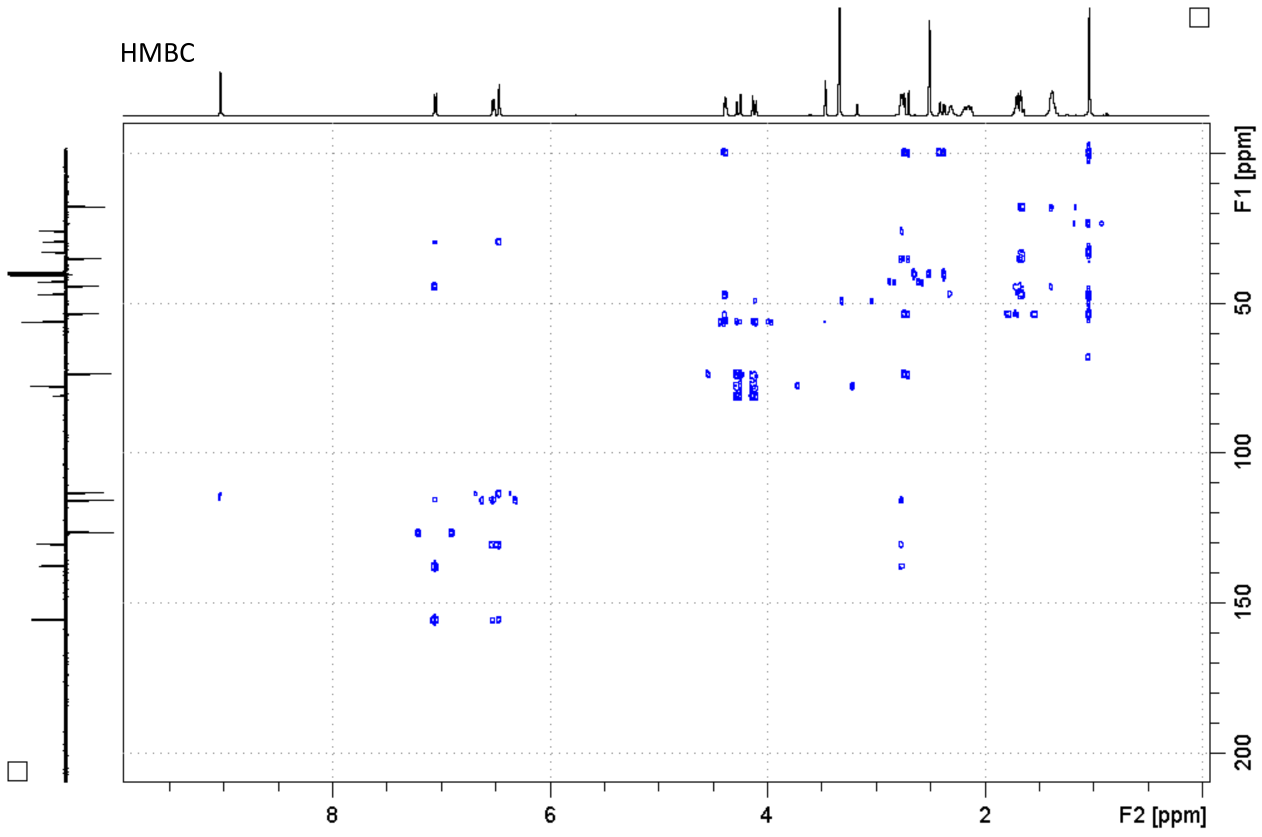
HMBC
F1 [ppm]
F2 [ppm]

NOESY

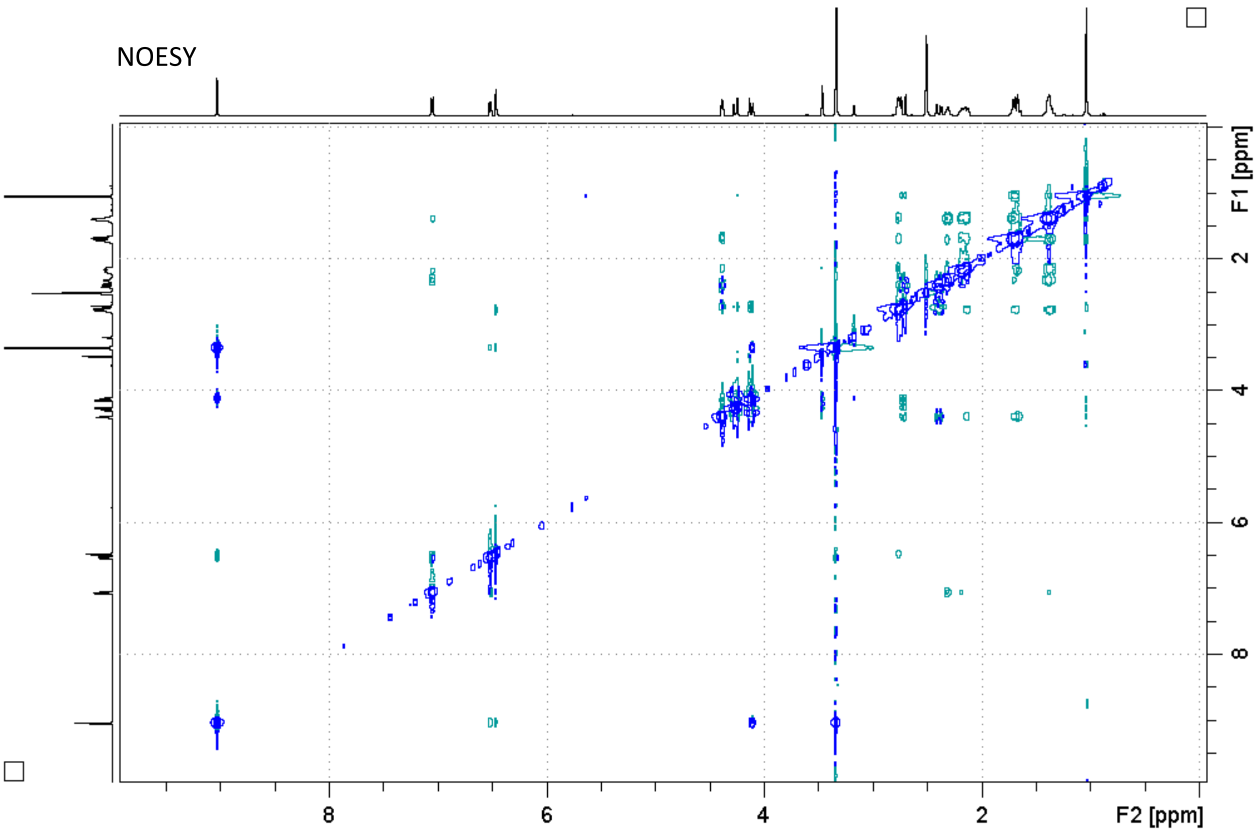

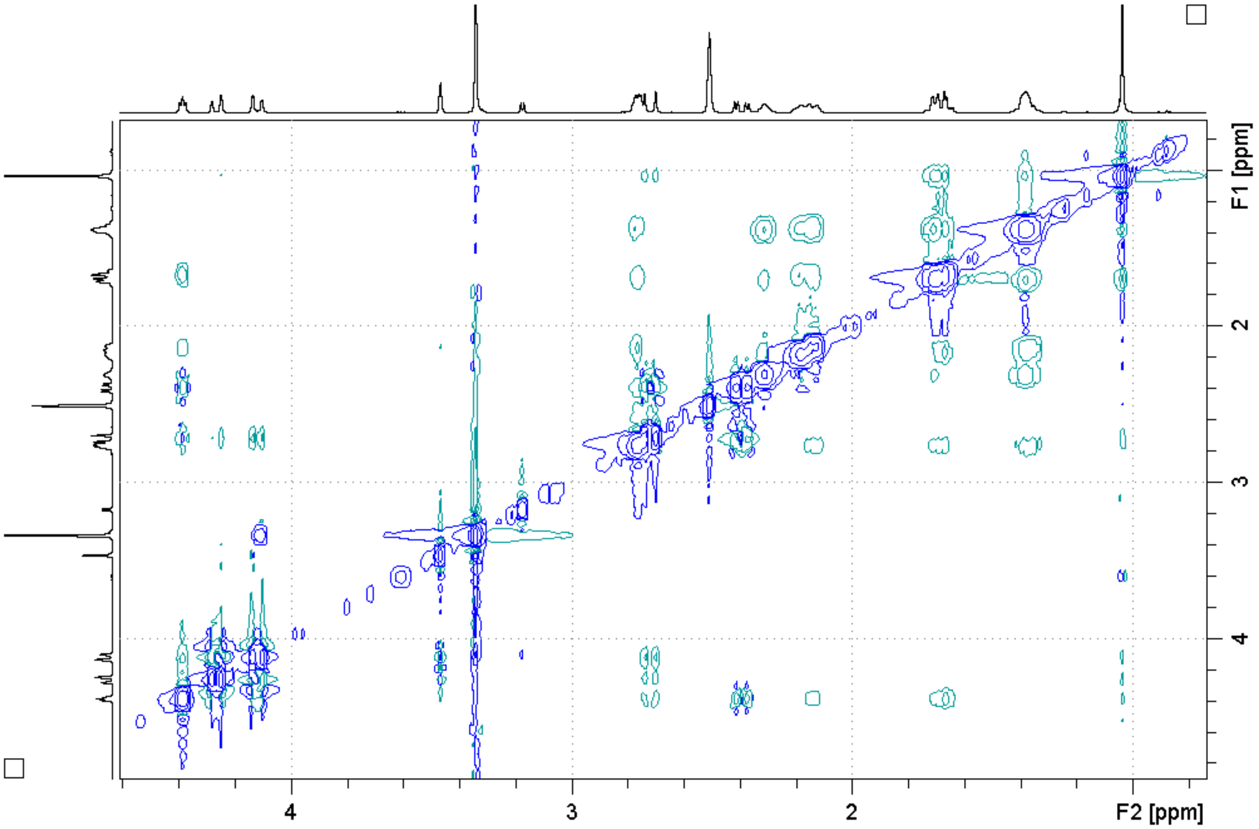

18

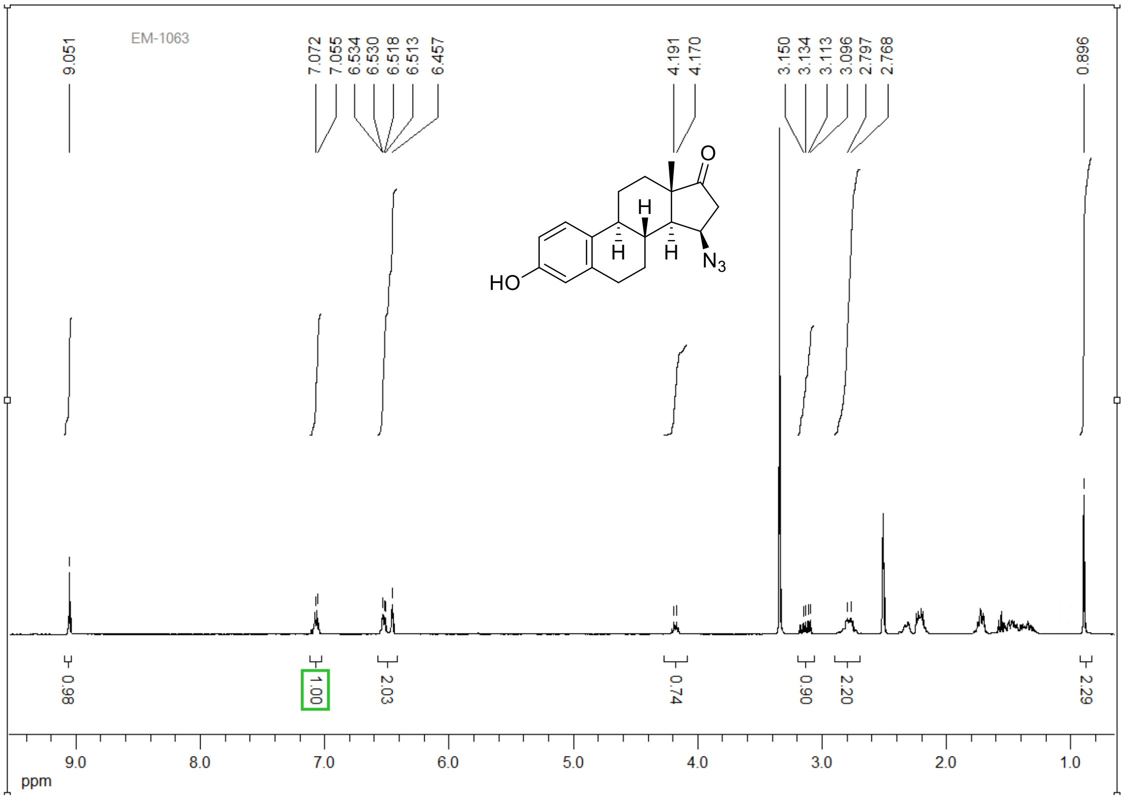
EM-1063
9.051
7.072
7.055
6.534
6.530
6.518
6.513
6.457
4.191
4.170
3.150
3.134
3.113
3.096
2.797
2.768
0.896
HO
H
H
H
O
N3
0.98
1.00
2.03
0.74
0.90
2.20
2.29
9.0
8.0
7.0
6.0
5.0
4.0
3.0
2.0
1.0
ppm

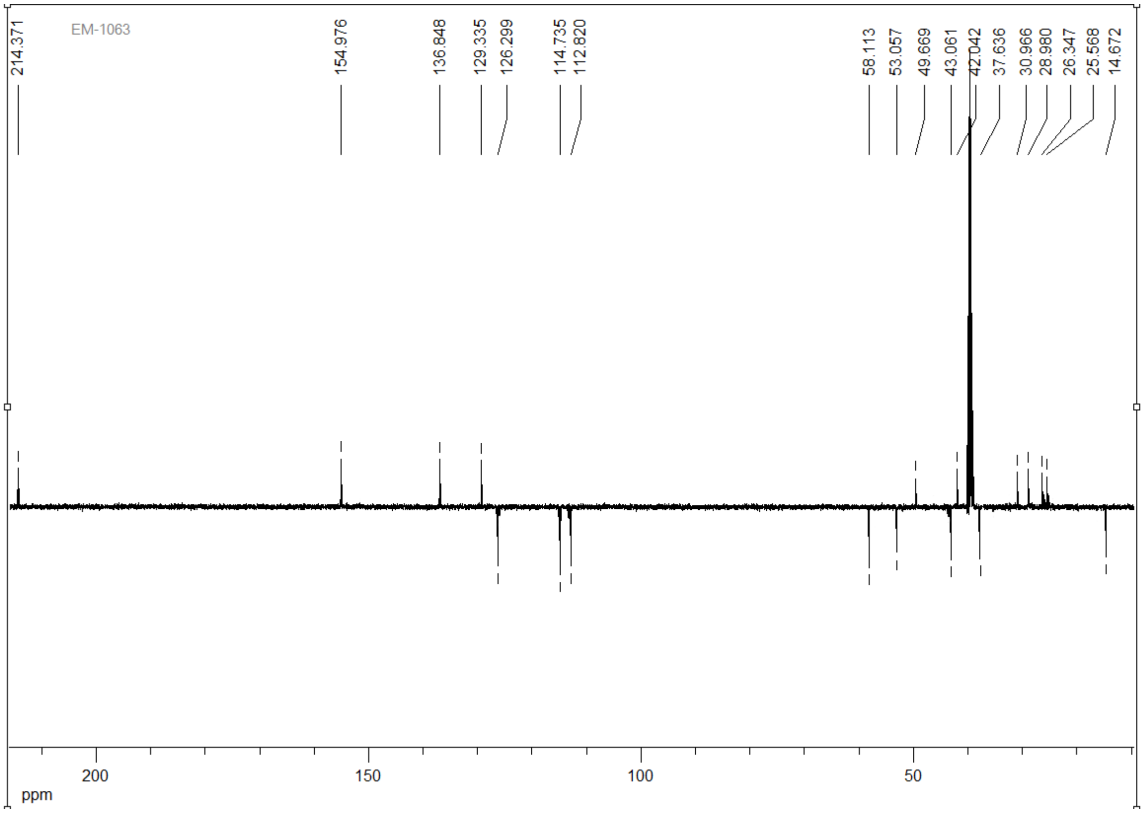
EM-1063
214.371
154.976
136.848
129.335
126.299
114.735
112.820
58.113
53.057
49.669
43.061
42.042
37.636
30.966
28.980
26.347
25.568
14.672
200
150
100
50
ppm

20

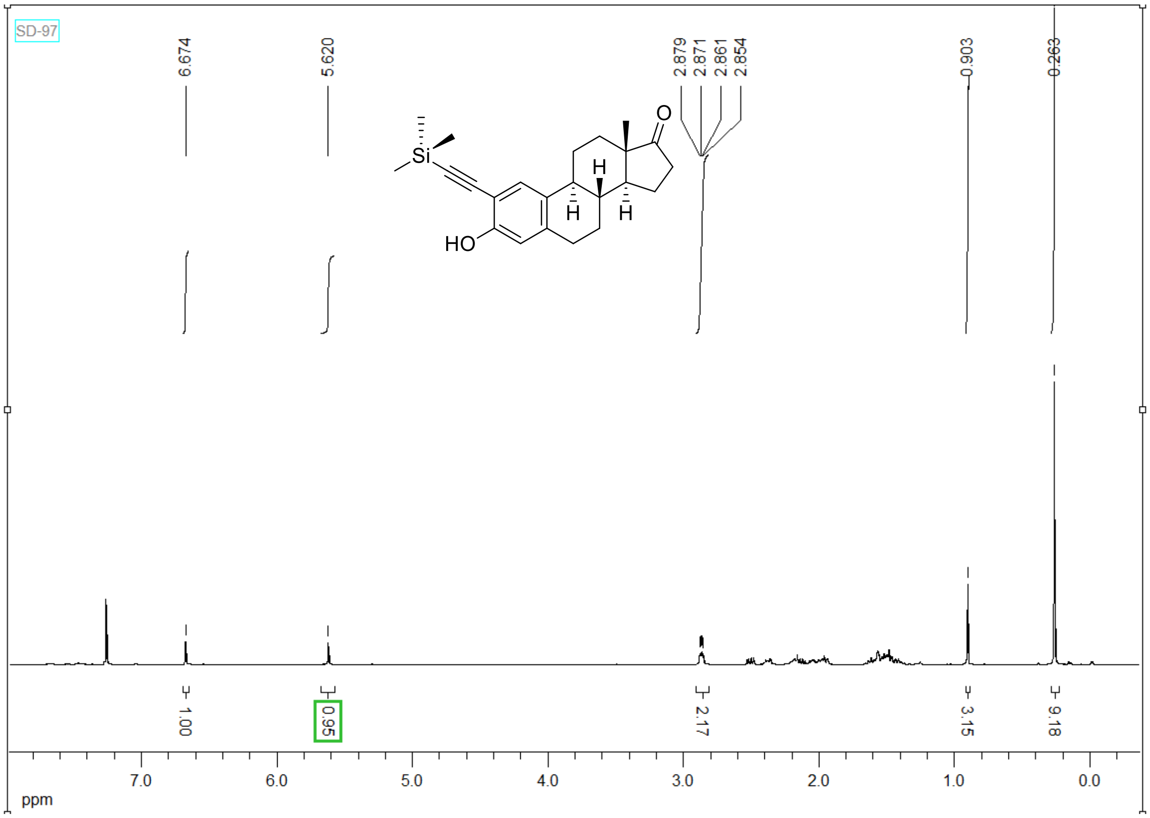
SD-97
6.674
5.620
2.879
2.871
2.861
2.854
0.903
0.263
Si
HO
O
H
H
H
1.00
0.95
2.17
3.15
9.18
7.0
6.0
5.0
4.0
3.0
2.0
1.0
0.0
ppm

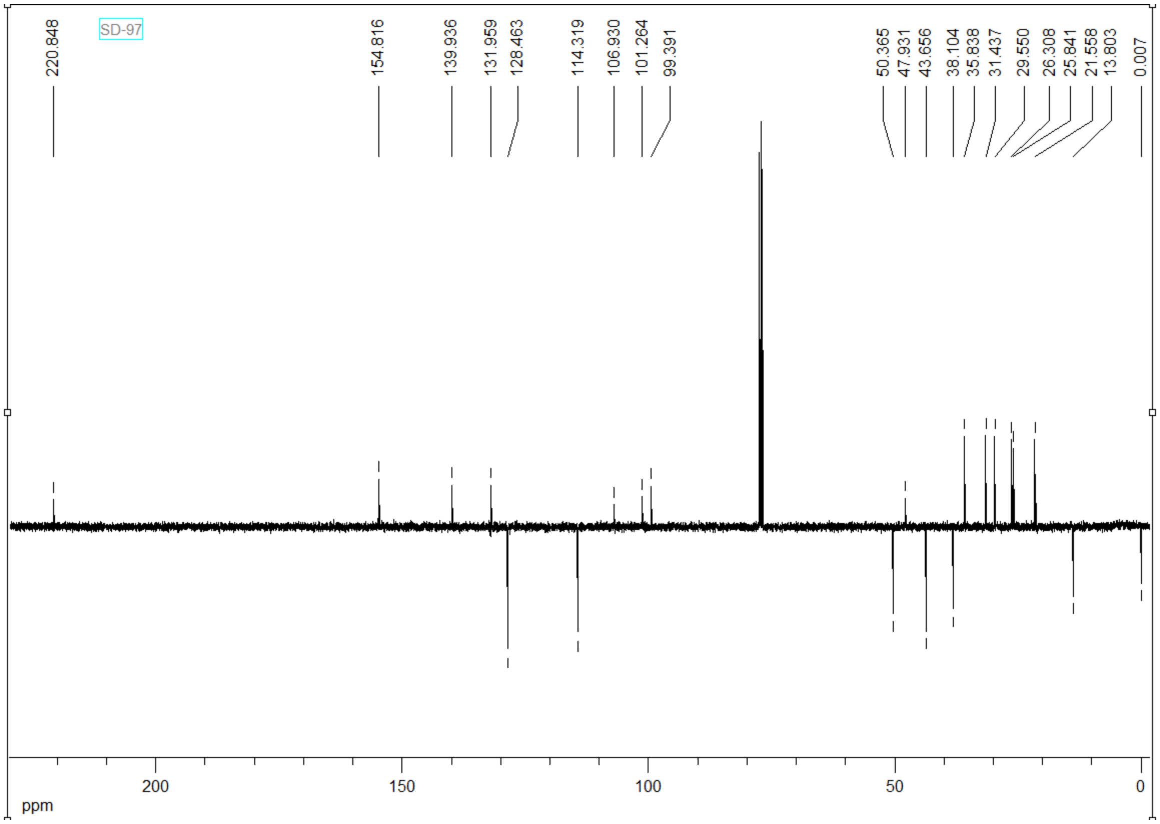
220.848
SD-97
154.816
139.936
131.959
128.463
114.319
106.930
101.264
99.391
50.365
47.931
43.656
38.104
35.838
31.437
29.550
26.308
25.841
21.558
13.803
0.007
200
150
100
50
0
ppm

22

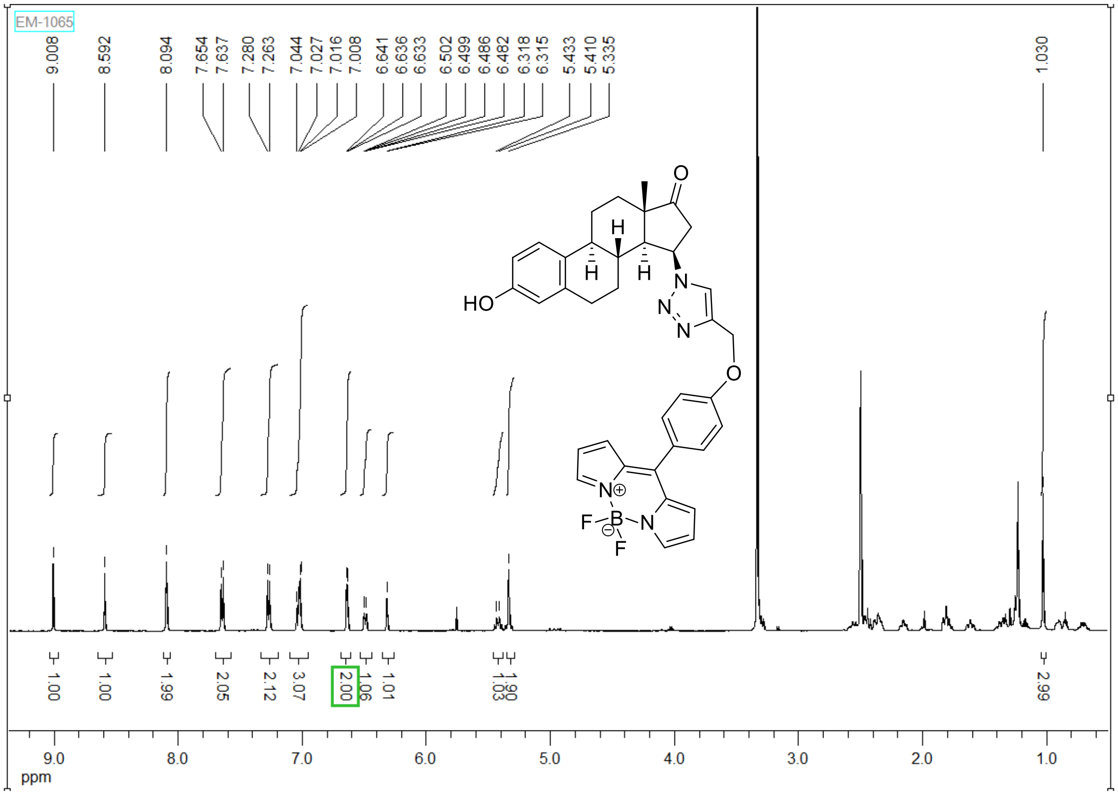

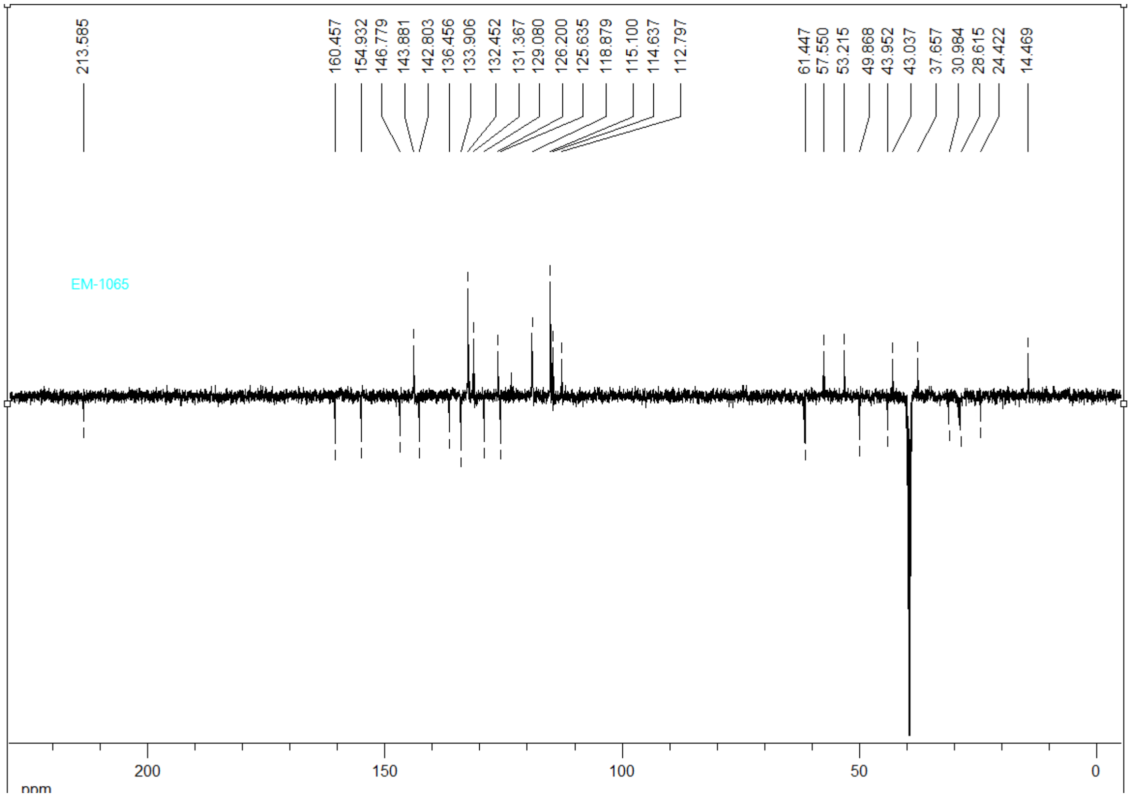

23

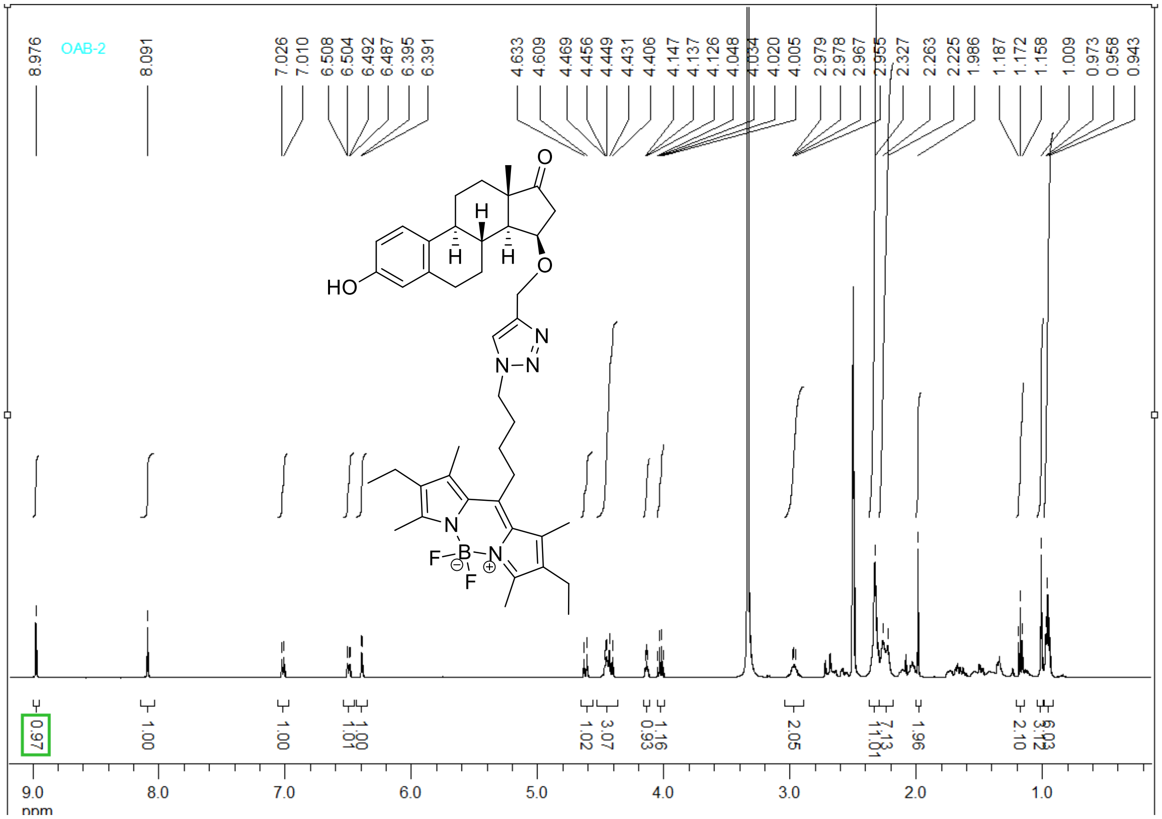

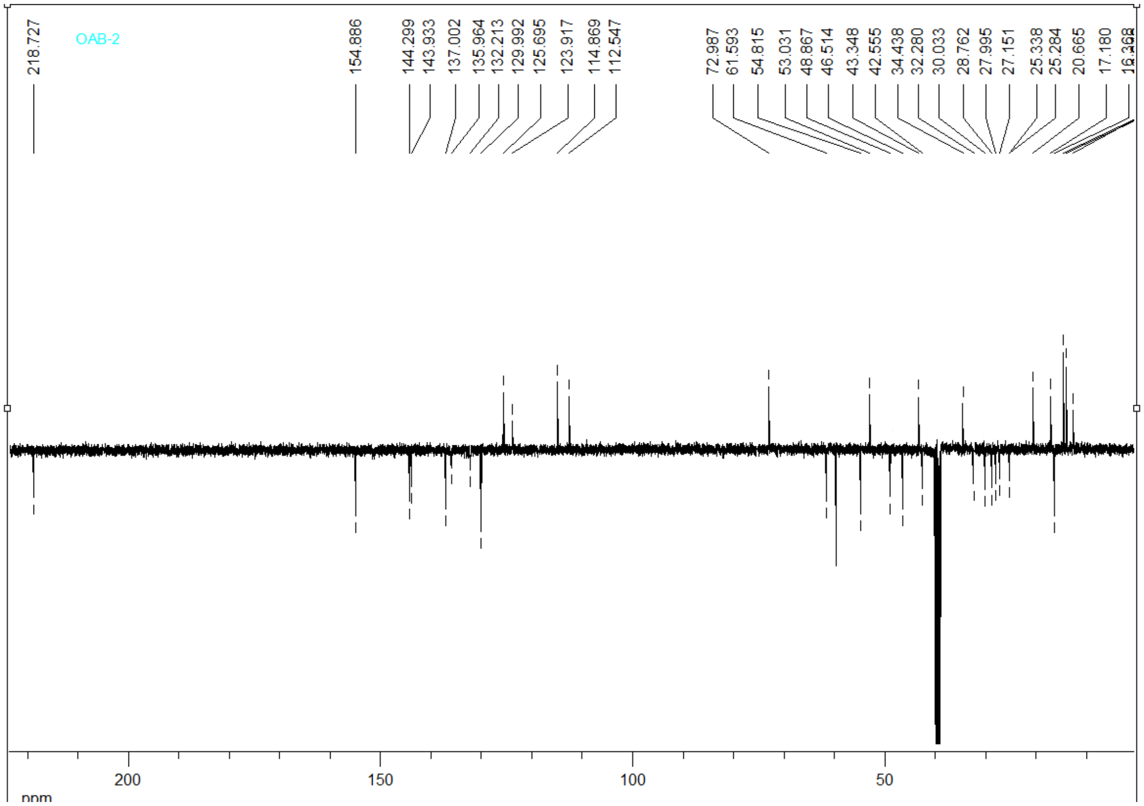

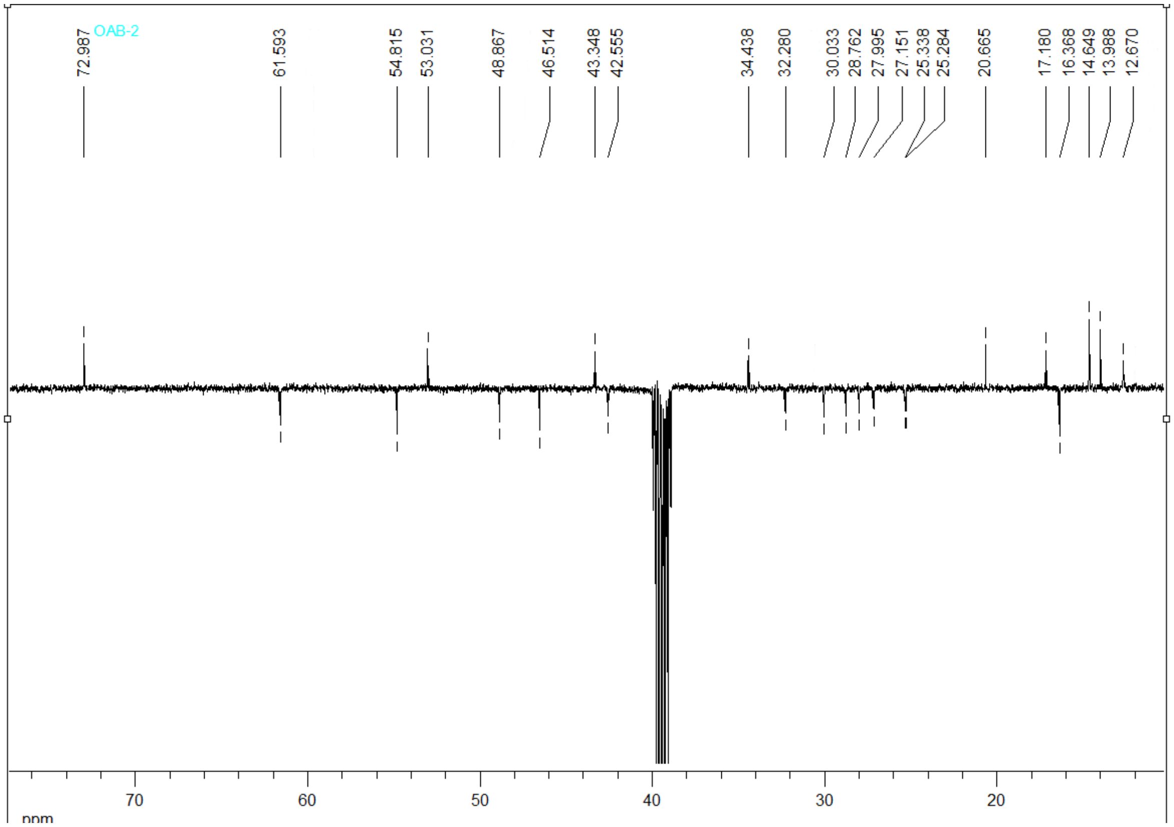

OAB-2
72.987
61.593
54.815
53.031
48.867
46.514
43.348
42.555
34.438
32.280
30.033
28.762
27.995
27.151
25.338
25.284
20.665
17.180
16.368
14.649
13.988
12.670
70
60
50
40
30
20
ppm

24

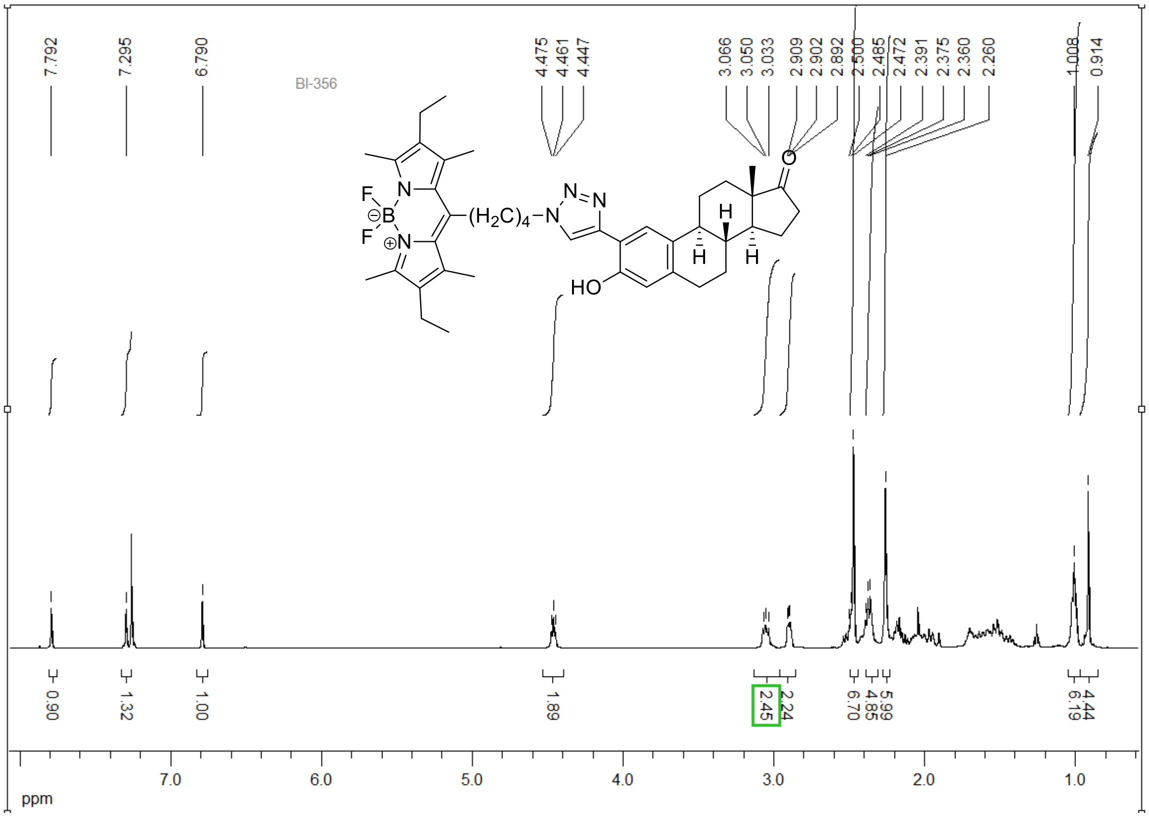
BI-356
7.792
7.295
6.790
4.475
4.461
4.447
3.066
3.050
3.033
2.909
2.902
2.892
2.500
2.485
2.472
2.391
2.375
2.360
2.260
1.008
0.914
0.90
1.32
1.00
1.89
2.45
2.24
6.70
4.85
5.99
6.19
4.44
ppm
7.0
6.0
5.0
4.0
3.0
2.0
1.0

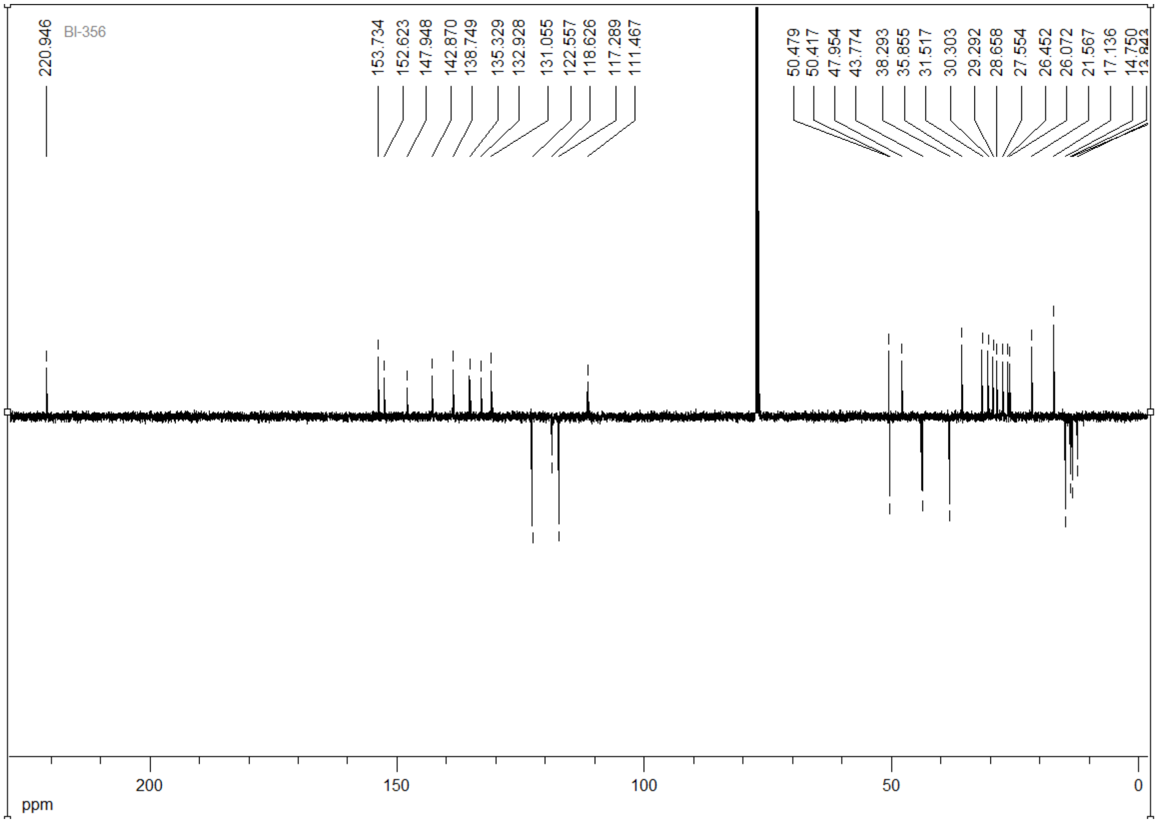
BI-356
220.946
153.734
152.623
147.948
142.870
138.749
135.329
132.928
131.055
122.557
118.626
117.289
111.467
50.479
50.417
47.954
43.774
38.293
35.855
31.517
30.303
29.292
28.658
27.554
26.452
26.072
21.567
17.136
14.750
ppm
200
150
100
50
0

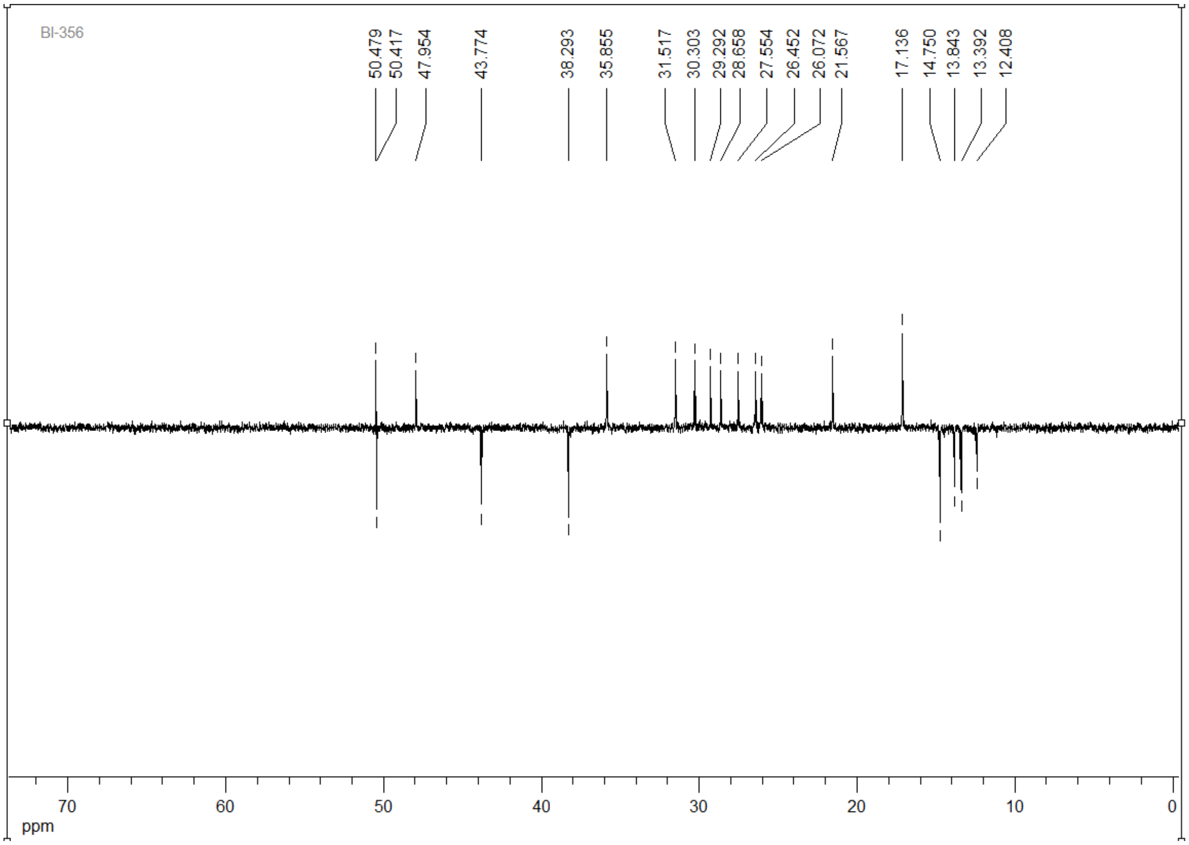
BI-356
50.479
50.417
47.954
43.774
38.293
35.855
31.517
30.303
29.292
28.658
27.554
26.452
26.072
21.567
17.136
14.750
13.843
13.392
12.408
70
60
50
40
30
20
10
0
ppm

Mass spectra

22

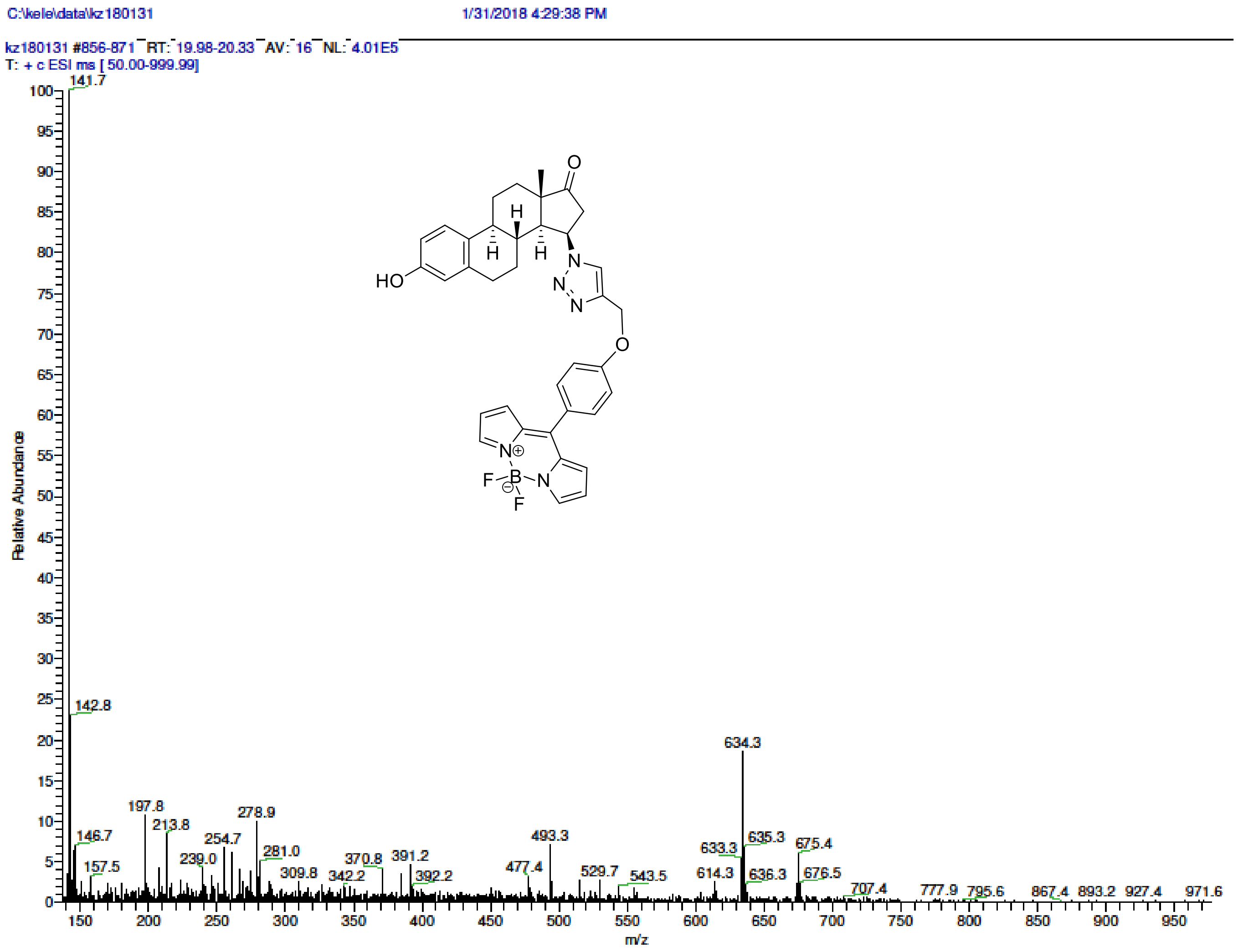

23

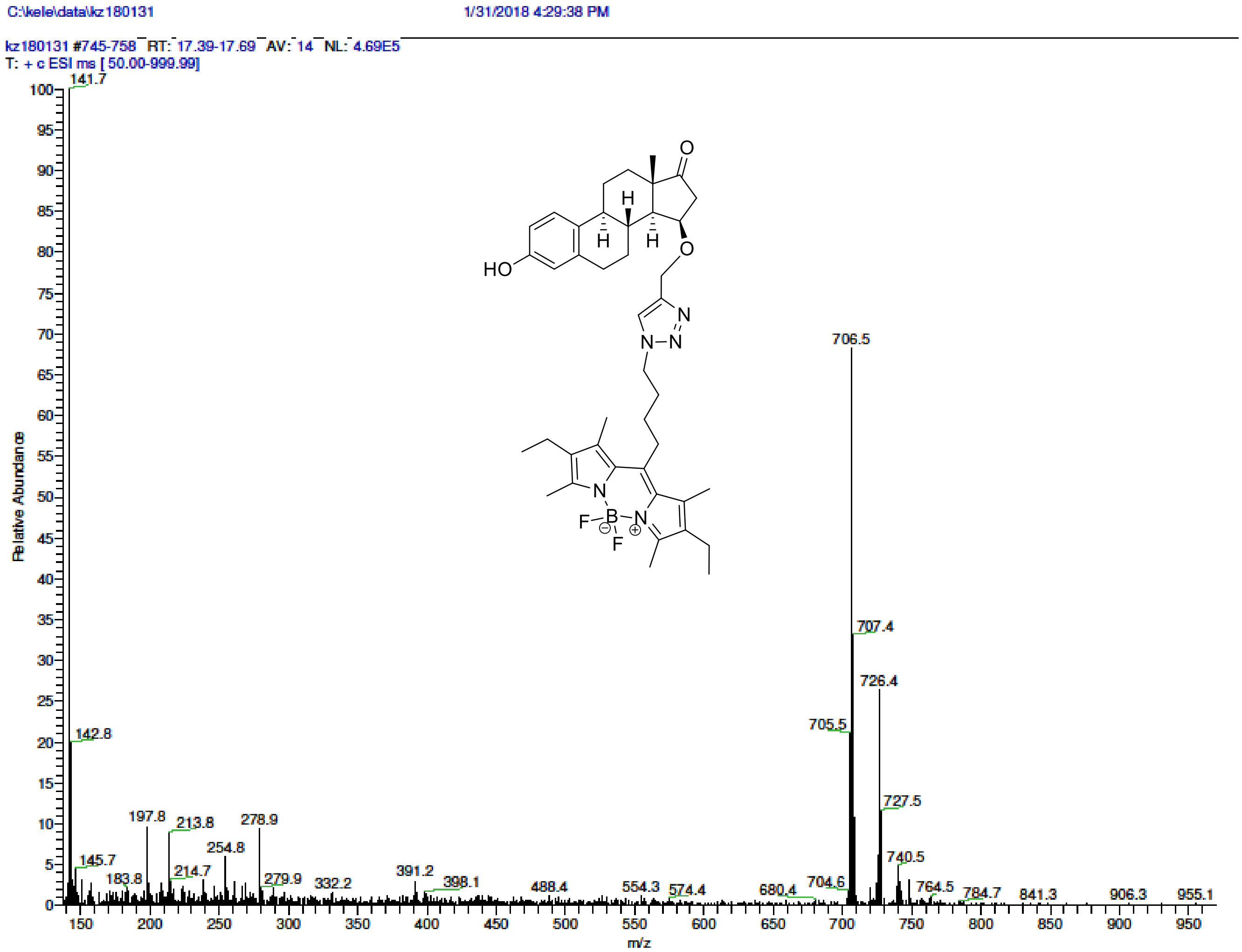
C:\kele\data\kz180131
1/31/2018 4:29:38 PM
kz180131 #745-758 RT: 17.39-17.69 AV: 14 NL: 4.69E5
T: + c ESI ms [50.00-999.99]
Relative Abundance
m/z
141.7
142.8
145.7
183.8
197.8
213.8
214.7
254.8
278.9
279.9
332.2
391.2
398.1
488.4
554.3
574.4
680.4
704.6
705.5
706.5
707.4
726.4
727.5
740.5
764.5
784.7
841.3
906.3
955.1
HO
H
O
N
B
F

24

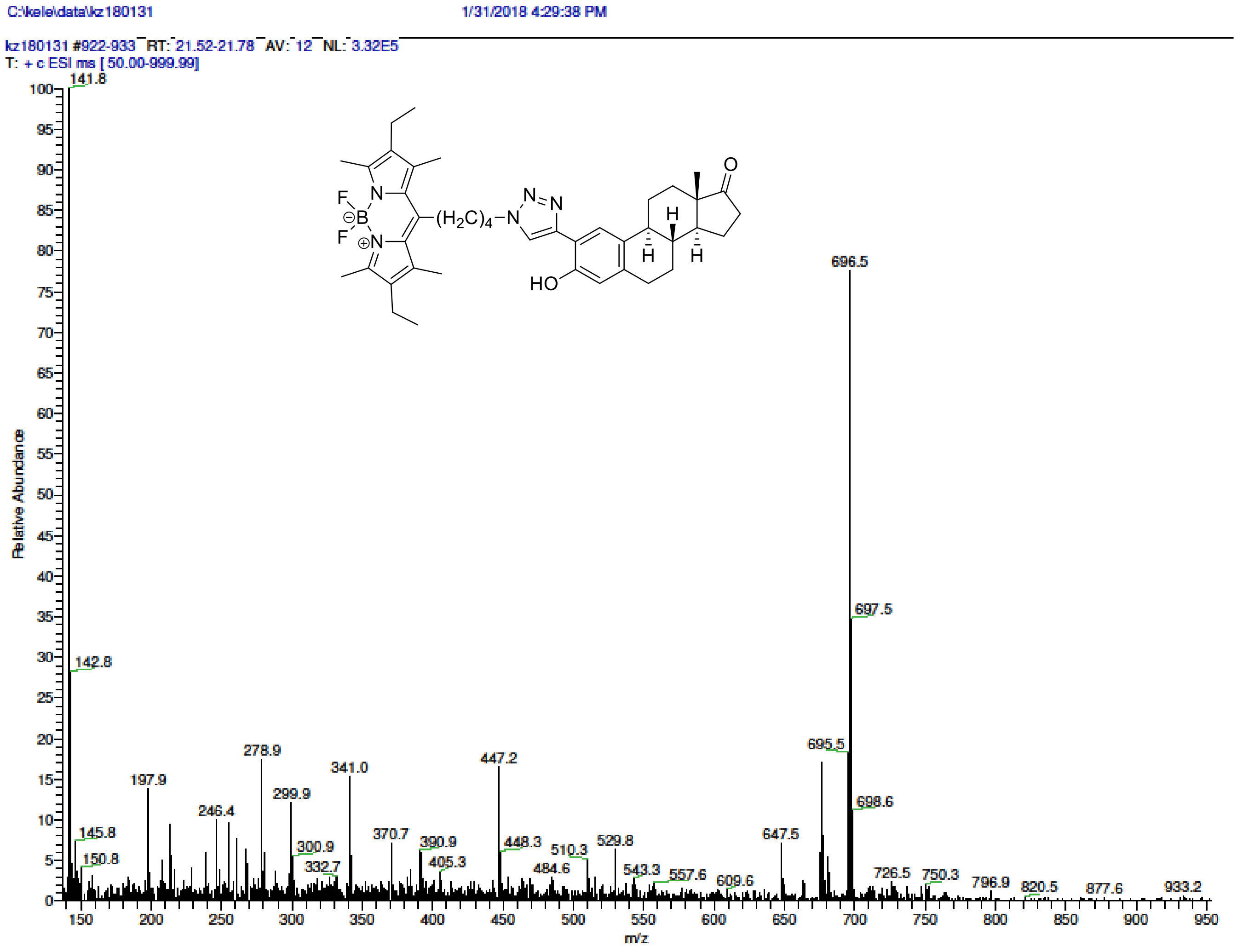
C:\kele\data\kz180131
1/31/2018 4:29:38 PM
kz180131 #922-933 RT: 21.52-21.78 AV: 12 NL: 3.32E5
T: + c ESI ms [50.00-999.99]
Relative Abundance
m/z
141.8
142.8
145.8
150.8
197.9
246.4
278.9
299.9
300.9
332.7
341.0
370.7
390.9
405.3
447.2
448.3
484.6
510.3
529.8
543.3
557.6
609.6
647.5
695.5
696.5
697.5
698.6
726.5
750.3
796.9
820.5
877.6
933.2
F
B
F
N
N
(H2C)4
N
N
N
HO
H
H
H
O
